# Meta-analysis with Jeffreys priors: Empirical frequentist properties

**DOI:** 10.1017/rsm.2024.2

**Published:** 2025-03-12

**Authors:** Maya B. Mathur

**Affiliations:** Quantitative Sciences Unit and Department of Pediatrics, Stanford University, Palo Alto, CA, USA

**Keywords:** meta-analysis, bayesian, simulation study, Firth correction, bayesian methods, small-sample estimation, simulations

## Abstract

In small meta-analyses (e.g., up to 20 studies), the best-performing frequentist methods can yield very wide confidence intervals for the meta-analytic mean, as well as biased and imprecise estimates of the heterogeneity. We investigate the frequentist performance of alternative Bayesian methods that use the invariant Jeffreys prior. This prior has the usual Bayesian motivation, but also has a purely frequentist motivation: the resulting posterior modes correspond to the established Firth bias correction of the maximum likelihood estimator. We consider two forms of the Jeffreys prior for random-effects meta-analysis: the previously established “Jeffreys1” prior treats the heterogeneity as a nuisance parameter, whereas the “Jeffreys2” prior treats both the mean and the heterogeneity as estimands of interest. In a large simulation study, we assess the performance of both Jeffreys priors, considering different types of Bayesian estimates and intervals. We assess point and interval estimation for both the mean and the heterogeneity parameters, comparing to the best-performing frequentist methods. For small meta-analyses of binary outcomes, the Jeffreys2 prior may offer advantages over standard frequentist methods for point and interval estimation of the mean parameter. In these cases, Jeffreys2 can substantially improve efficiency while more often showing nominal frequentist coverage. However, for small meta-analyses of continuous outcomes, standard frequentist methods seem to remain the best choices. The best-performing method for estimating the heterogeneity varied according to the heterogeneity itself. Röver & Friede’s R package bayesmeta implements both Jeffreys priors. We also generalize the Jeffreys2 prior to the case of meta-regression.

## Highlights

- What is already known: The best-performing frequentist methods for random-effects meta-analysis can be highly imprecise in small meta-analyses, and can provide biased estimates of the heterogeneity.
- What is new: We conduct a large simulation study evaluating two forms of the Jeffreys prior for meta-analysis, which correspond to the Firth bias correction to the maximum likelihood estimator.
- Potential impact for *RSM* readers: For small meta-analyses of binary outcomes, the Jeffreys2 prior may offer advantages over standard frequentist methods for point and interval estimation for the mean parameter.

## 1 Introduction

Standard random-effects meta-analysis involves estimating the heterogeneity of studies’ population effects (e.g., their standard deviation) and obtaining an inverse-variance-weighted estimate of the meta-analytic mean, in which studies’ weights depend on the estimated heterogeneity.^1^ Commonly used methods to estimate the heterogeneity include semiparametric method-of-moments estimators^1^ ^–^ ^5^ and parametric likelihood-based estimators.^1^ ^,^ ^6^ The theoretical justification for these methods relies on asymptotics, yet in some scientific disciplines, the majority of meta-analyses include a relatively small number of studies. Meta-analyses of healthcare interventions in the Cochrane Database for Systematic Reviews include a median of only 3 studies (75th percentile: 6, 90th percentile: 10).^7^ In psychology, meta-analyses published in *Psychological Bulletin* include a median of 12 studies, though some meta-analyses are much larger (75th percentile: 33, 90th percentile: 76).^8^ ^,^ ^9^

On the one hand, previous simulation studies indicate that even in very small meta-analyses (here defined as those with 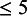 studies), many existing methods provide nearly unbiased point estimates for the meta-analytic mean, termed 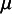 .^10^ On the other hand, confidence intervals that are based on asymptotic normality (e.g., Wald intervals) can have less than nominal coverage in small meta-analyses ( 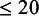 studies), and coverage can decline further in very small meta-analyses.^7^ ^,^ ^11^ ^,^ ^12^ Using Hartung–Knapp–Sidik–Jonkman’s (HKSJ) method to adjust standard errors^13^ ^,^ ^14^ can provide better-calibrated intervals in many settings, though existing simulation studies have yielded somewhat mixed findings regarding whether these intervals consistently achieve nominal coverage.^7^ ^,^ ^11^ ^,^ ^12^ ^,^ ^15^ ^–^ ^17^ Moreover, such intervals can be extremely wide for meta-analyses of typical sample sizes.^15^ ^–^ ^18^ For example, even when the true heterogeneity is zero, moments estimators with HKSJ standard errors yielded 95% confidence intervals with average widths of approximately 4–5 in simulated meta-analyses of 5 studies.^18^ This suggests that for a point estimate of 0.5 on the standardized mean difference scale, a typical confidence interval would be approximately 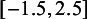 , which is so wide that it might be considered uninformative. Additionally, standard point estimates for the heterogeneity can be substantially biased and imprecise in small meta-analyses.^7^ ^,^ ^11^ Many existing simulation studies on heterogeneity estimation do not seem to have evaluated the coverage or width of confidence intervals for the heterogeneity^11^ (but see Viechtbauer (2007)^19^).

In this paper, we investigate the frequentist performance of alternative Bayesian methods that use the invariant Jeffreys prior.^20^ In general, Bayesian estimation proceeds by specifying a prior on the unknown parameters and obtaining the posterior of those parameters, given the observed data. This essentially involves updating the prior based on the likelihood of the observed data.^21^ Various types of point estimates and credible intervals can then be obtained from the posterior. For an arbitrary distribution with unknown parameters 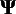 and expected Fisher information 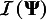 , the Jeffreys prior is proportional to 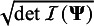 .^20^ An original motivation for this prior was its invariance to transforming the parameters,^20^ a property that does not hold for all priors.^22^ ^,^ ^23^ ^,^ ^i^ For example, letting 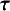 denote the standard deviation of studies’ population effects, the Jeffreys prior on 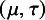 is the same as the Jeffreys prior on 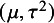 , so the resulting posterior estimates and intervals would not depend on the analyst’s arbitrary choice of parameterization. This desirable property has led some to describe the Jeffreys prior as “noninformative,” though we agree with others’ critiques of this term.^24^ ^,^ ^25^

An interesting, underappreciated property of the Jeffreys prior is that the resulting posterior can alternatively be motivated from a solely frequentist perspective.^26^ In particular, it is well-known that the maximum likelihood (ML) estimate has an 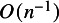 bias, essentially due to the curvature of the score function.^26^ Firth (1993)^26^ showed that for exponential family distributions, an appropriate penalty on the likelihood to correct this bias coincides with estimation under the Jeffreys prior. This is essentially because the Jeffreys prior introduces a bias in the score function that compensates for the bias due to its curvature.^26^ In particular, the posterior mode under this prior can be viewed in frequentist terms as a bias-corrected ML estimate; consequently, the posterior mode under the Jeffreys prior has sometimes been termed the “Firth correction.” The Firth correction has demonstrated success in a number of frequentist estimation problems, and is used fairly often for logistic regression.^26^ ^–^ ^29^

Given the Jeffreys prior’s effectiveness as a bias-correction method in small samples, it seems plausible that using this prior in small meta-analyses might improve point and interval estimation. Bodnar et al. (2016, 2017)^15^ ^,^ ^30^ derived the Jeffreys prior on the heterogeneity 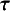 alone (i.e., holding the mean 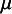 constant), an approach that may be optimal if 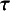 is strictly a nuisance parameter.^25^ Their simulations suggested that, along with an independent flat prior on 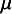 , the resulting credible intervals may have better frequentist coverage than existing frequentist methods.^15^ We term this prior “Jeffreys1” because it is the prior with respect to a single parameter. Kosmidis et al. (2017)^31^ independently derived a penalized likelihood correction that is equivalent to the single-parameter Jeffreys prior on 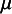 alone; that is, treating 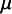 rather than 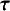 as a nuisance parameter. This penalization is closely related to the restricted ML (REML) estimator of 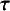 .^31^

In this paper, we consider the Jeffreys1 prior along with the two-parameter Jeffreys prior on both 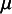 and 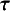 . To the best of our knowledge, the latter has not appeared in the published literature on meta-analysis. We consider this prior, termed “Jeffreys2”, for several reasons. First, while the mean parameter is often of primary interest in meta-analysis, the heterogeneity should generally also be estimated and reported, so it may not be optimal to treat 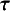 as a nuisance parameter.^32^ Second, in other small-sample estimation problems, multiparameter Jeffreys priors that include scale parameters (e.g., the dispersion parameter in exponential-family models) have been proposed and have good empirical properties.^26^ ^,^ ^28^ ^,^ ^33^ (We return to this issue in Section 3.3.) In the context of adjusting for *p*-hacking in meta-analyses by meta-analyzing only a truncated part of the random-effects distribution, we recently found that a Jeffreys prior on 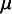 and 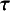 performed considerably better than ML,^34^ whose performance is remarkably poor for truncated distributions in general.^28^ ^,^ ^35^ Third, as we will discuss, the shape of the Jeffreys2 prior suggests it might provide more precise intervals than the Jeffreys1 prior. Whether Jeffreys2 credible intervals show nominal frequentist coverage, and whether point estimation for 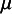 and 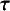 performs well, are open questions.

Previous simulation studies of Jeffreys priors in meta-analysis have provided promising preliminary results, but do have limitations. Those simulations investigated only the Jeffreys1 prior, but not Jeffreys2, and have considered point and interval estimation for 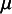 , but not 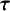 .^15^ In this paper, we present a simulation study comparing the frequentist properties of point and interval estimation for both 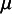 and 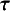 under the Jeffreys1 and Jeffreys2 priors, as well as several of the best-performing frequentist methods. Using a simulation design that closely paralleled a recent, extensive simulation study by Langan et al. (2019)^7^, we substantially expanded on the range of comparison methods and simulation scenarios used in previous simulation studies of the Jeffreys1 prior. Previous simulations regarding the Jeffreys1 prior considered only posterior means for point estimation,^15^ whereas the aforementioned bias-correction properties specifically apply to posterior modes. This may be especially relevant for point estimation of 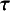 , whose posterior is highly asymmetric. We therefore consider three types of Bayesian point estimates (the posterior mode, mean, and median) as well as two types of credible intervals (central and shortest). Our simulations include the best-performing methods in Langan et al.’s (2019)^7^ simulation study, along with several other methods whose theoretical properties suggest they might also perform well, such as exact intervals^18^ and intervals based on the profile likelihood.^6^

This paper is organized as follows. We briefly review existing moments and likelihood-based estimators for random-effects meta-analysis (Section 2), all of which have been covered in more detail elsewhere.^6^ ^,^ ^18^ ^,^ ^36^ We also briefly review existing simulation results regarding these methods (Section 2.4). We review the established form of the Jeffreys1 prior^15^ and derive the form of the Jeffreys2 prior; we then discuss posterior estimation under both priors (Section 3). We present the simulation study (Section 4) and a brief applied example (Section 5), and conclude with a general discussion.

## 2 Existing frequentist methods

### 2.1 Method-of-moments estimators

Moments estimators for meta-analysis are semiparametric; they involve specifying only the first two moments of the distribution of population effects, namely 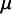 and 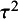 . Because these methods do not require specifying the higher moments, they do not requiring assuming that population effects are normal. Specifically, consider *k* studies whose population effects, 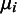 , have expectation 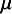 and variance 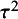 . These two moments are the usual meta-analytic estimands of interest. Let 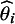 and 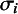 respectively denote the point estimate and standard error of the *i*th study, such that 
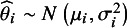
 holds approximately. The within-study standard errors 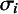 are generally treated as fixed and known.

For a given estimate of the heterogeneity variance, 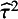 , the estimated marginal variance of 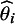 is 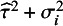 . The uniformly minimum variance unbiased estimator (UMVUE) of 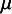 arises from weighting studies by the inverse of their estimated marginal variances,^6^ denoted 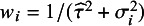 : 

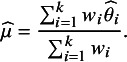

The various moments estimators are distinguished by their estimators for 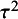 , and hence the form of the weights 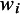 . Detailed reviews^7^ ^,^ ^36^ ^,^ ^37^ and original papers on these approaches are available, so here we summarize briefly. Moments estimators for 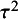 are based on the generalized Q-statistic: (1)

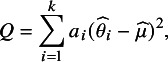

where the form of the coefficients, 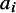 , differs across moments estimators. For example, the traditional Dersimonian–Laird estimator (DL)^1^ sets 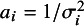 . The two-step DL estimator (DL2)^2^ instead sets 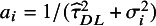 , where 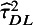 is an initial estimate obtained using the DL estimator. The Paule–Mandel (PM)^3^ ^,^ ^4^ estimator can be viewed as a limiting case of DL2, involving iteration over the estimates 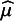 and 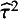 until convergence. This estimator is also equivalent to the empirical Bayes estimator.^5^ In general terms, empirical Bayes estimation uses the observed data to estimate the parameters of the Bayesian prior, rather than specifying the prior independently of the data.^21^ In the context of meta-analysis, the empirical Bayes estimator essentially estimates the distribution of population effects by their posterior means, with the prior determined empirically.^5^

### 2.2 Likelihood-based estimators

In contrast to moments estimators, commonly used likelihood-based estimators assume that the population effects, 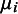 , arise independently from the distribution 
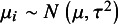
 . Thus, the marginal distribution of studies’ point estimates, 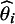 , is 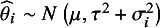 . We denote the *k*-vector of point estimates as 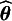 . Letting 
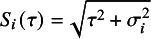
 be the true marginal standard deviation of the *i*th study, the joint likelihood is: (2)

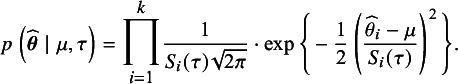

The standard ML estimator for 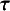 is obtained as usual by solving 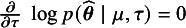 , whose solution depends on 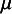 .^6^ Since this estimator does not take into account the loss in degrees of freedom due to the additional estimation of 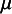 itself, the resulting estimate is often negatively biased.^6^ This issue motivates REML estimation, which can improve upon ML estimation by transforming the log-likelihood to remove the parameter 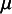 .^6^

### 2.3 Interval estimation

A simple Wald confidence interval can be obtained by assuming 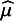 is normally distributed, which holds asymptotically in *k* by standard ML properties. If the weights 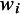 are treated as known rather than estimated, we have 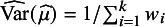 . A Wald 95% confidence interval is: 

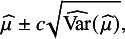

where 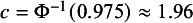 is the critical value of the standard normal distribution. However, Wald intervals exhibit substantial under-coverage for small meta-analyses, both because the normal approximation holds only asymptotically and because the approximation 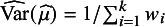 does not account for the estimation of 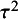 .^7^ ^,^ ^11^ ^,^ ^12^ Wald intervals can also be constructed for 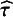 , but exhibit similarly poor performance.^19^ We therefore do not further discuss Wald intervals, focusing instead on the better-performing alternatives discussed below.

Regarding interval estimation for 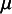 , the alternative HKSJ, sometimes called “Knapp–Hartung,” interval addresses the limitations of the Wald interval.^13^ ^,^ ^14^ This method more flexibly assumes that 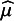 follows a *t* distribution and additionally rescales 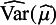 to account for the estimation of 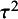 in the weights 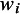 : 

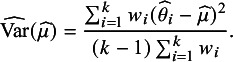

For 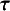 , improved intervals can be constructed using the chi-square distribution of the Q statistic, per Eq. (1).^19^ These “Q-profile” intervals substantially outperform Wald intervals.^19^ For both 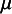 and 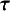 , ML profile intervals can also be constructed in the usual way.^6^

An interesting, relatively new approach provides exact rather than asymptotic intervals and is theoretically guaranteed to provide more than nominal coverage, under the assumption of normal population effects.^18^ This method essentially involves inverting exact tests. Other parametric methods provide finite-sample corrections to the likelihood ratio test statistic; these include Skovgaard’s second-order correction and Bartlett’s correction.^38^ ^–^ ^40^ These methods can improve upon basic likelihood methods for hypothesis testing,^40^ but Skovgaard’s second-order correction was not designed for interval estimation and can be numerically unstable in this context.^31^ Interval estimation with Bartlett’s correction is possible,^41^ but is not implemented in existing software (I. Visser, personal communication, 8 July 2024).^42^ ^,^ ^43^ Because our focus is on interval estimation rather than testing, our simulations do not include Skovgaard’s or Bartlett’s corrections. Finally, various parametric or nonparametric resampling methods can be used to obtain bootstrapped confidence intervals.^19^ ^,^ ^43^ ^,^ ^44^ Nonparametric resampling can be conducted by resampling rows with replacement, after which one can obtain simple percentile bootstrap intervals or bias-corrected and accelerated (BCa) intervals, among many other types of bootstrap intervals.^45^ ^,^ ^46^ The BCa confidence corrects for bias and skewness in the bootstrapped sampling distribution, which we speculate could be helpful when estimating the sampling distribution of 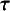 . The BCa bootstrap has performed relatively well for certain meta-analytic estimators that are functions of 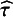 .^47^ However, bootstrapping is an asymptotic procedure whose finite-sample performance typically must be assessed through simulations.

### 2.4 Existing simulations comparing these methods

Langan et al. (2017)^11^ provide an excellent systematic review of simulation studies for different heterogeneity estimators.^7^ Briefly, the DL estimator was negatively biased for 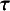 when heterogeneity was moderate to high, and the PM estimator was typically less biased.^11^ The reviewed studies do not appear to have assessed interval estimation for 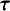 . Based on their own, more extensive simulation study, Langan et al. (2019)^7^ generally recommend REML, PM, or DL2 for heterogeneity estimation, along with HKSJ confidence intervals for 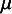 ; however, they recommend caution in interpreting heterogeneity estimates in small meta-analyses.

Langan et al.’s (2019)^7^ simulation study did not assess intervals based on the profile likelihood, bootstrapping, or the exact method; the latter was developed only recently. Regarding profile intervals, recommendations in the literature are inconsistent. A prominent paper stated that “the profile likelihood is a good method for computing confidence intervals”.^48^ One simulation study seemed to support this recommendation, finding that when the heterogeneity is greater than zero, profile likelihood intervals showed the closest to nominal coverage.^10^ On the other hand, another simulation study suggested that profile intervals often exhibited under-coverage for meta-analyses of only 5 studies.^39^ The originators of the exact method provide simulations suggesting that the resulting intervals are not substantially wider than those of existing methods, despite the method’s theoretical guarantee of at least nominal coverage.^18^ While our simulation study is primarily motivated by investigating the Jeffreys methods, a secondary contribution is to more extensively evaluate profile, bootstrap, and exact intervals. We now turn to establishing the theory for the Jeffreys1 and Jeffreys2 priors.

## 3 Bayesian methods using Jeffreys priors

### 3.1 The Jeffreys priors

Under the assumption of normal population effects, Bodnar et al. (2017)^15^ showed that the improper Jeffreys1 prior is: 

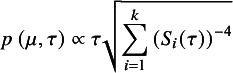

where, again, 
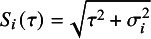
 . Since this prior is independent of 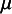 , it can be expressed as two independent priors on 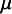 and 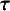 , where the prior on 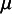 is uniform: (3)

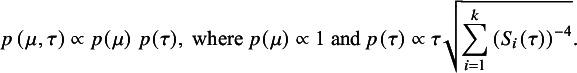



If 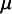 is treated as the only parameter of interest and 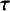 is considered a nuisance parameter, then the Jeffreys1 prior also coincides with the Berger–Bernardo reference prior.^30^ In general, the Berger–Bernardo prior for a given distribution is designed to be maximally “noninformative” in the sense of minimizing the amount of information provided by the prior and maximizing the amount of information provided by the data.^30^ ^,^ ^49^ Specifically, this prior maximizes the Kullback–Liebler divergence between the prior and the posterior.^49^

Regarding the Jeffreys2 prior, the joint likelihood in Eq. (2) implies that the entries of the expected Fisher information are: 

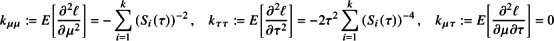

where 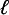 is the likelihood function. Therefore, the Jeffreys2 prior is 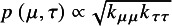 . (This result is straightforward to show directly, or alternatively can be viewed as a simple special case of the prior given in Mathur (2024).^34^ ^,^ ^ii^ This yields the improper two-parameter prior: 

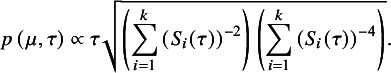



Like the Jeffreys1 prior, the Jeffreys2 prior can be expressed as: (4)

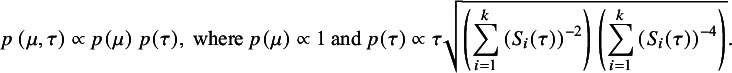



**Figure 1:**
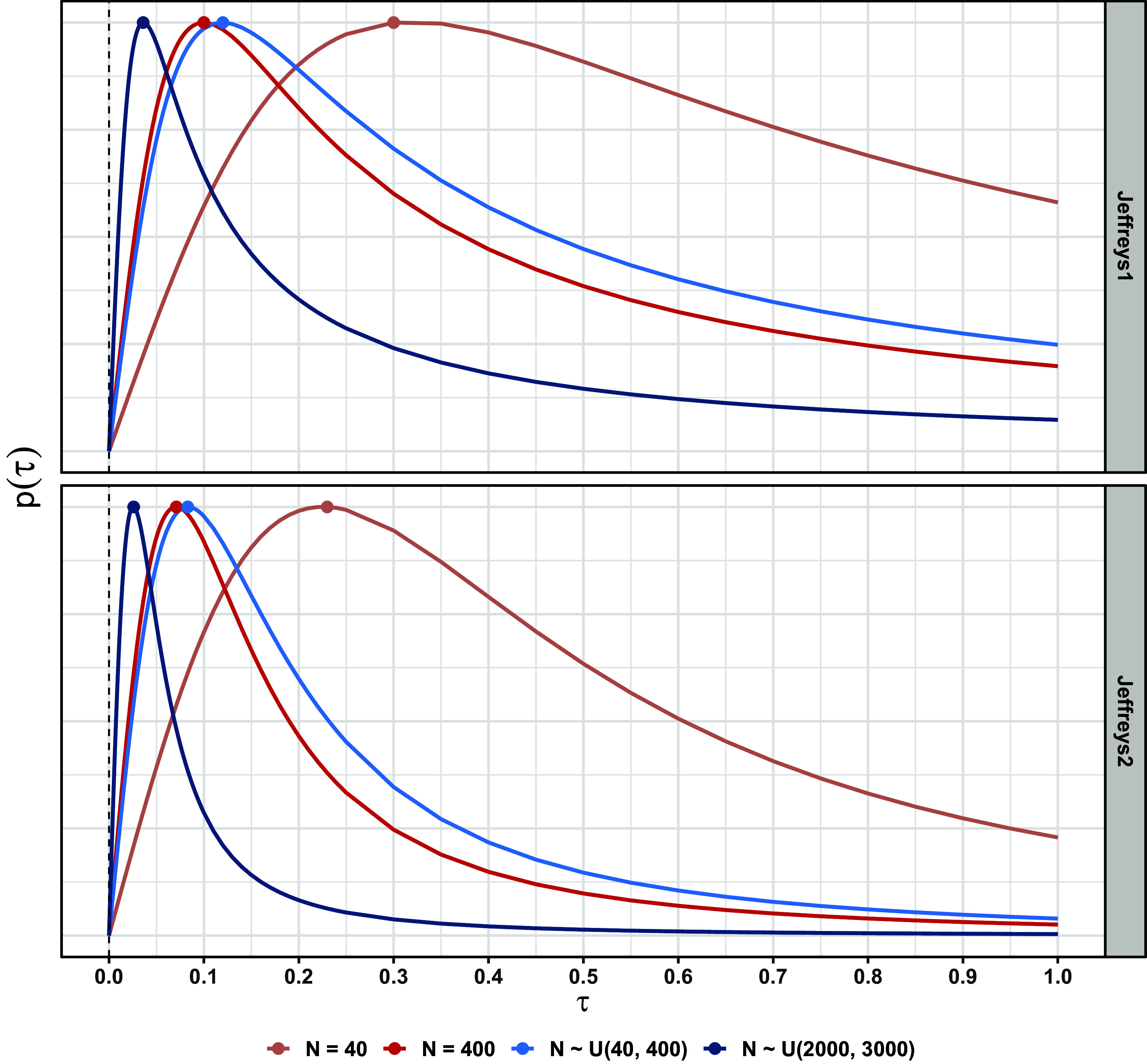
Priors for four simulated meta-analyses of standardized mean differences ( 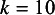 ), in which the within-study sample sizes (*N*) were generated from four possible distributions. Studies’ standard errors were estimated using Eq. (5) and, given the data-generation parameters, were approximately equal to 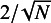 . Points are the maxima. The priors have been scaled to have the same maximum height.

To illustrate, Figure 1 shows both priors on 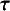 for four meta-analyses of standardized mean differences. The meta-analyses were simulated with studies’ sample sizes, *N*, arising from four different distributions. Although the magnitude of the priors will of course be affected by the number of studies *k*, their shape is minimally affected by *k*, so Figure 1 depicts the prior for meta-analyses with 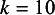 . Note that for each meta-analysis, the Jeffreys2 prior is somewhat narrower than the Jeffreys1 prior, suggesting that the former may provide narrower intervals; this hypothesis will be explored in more depth in the simulation study (Section 4). Both priors lead to proper posteriors if 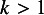 (see Bodnar (2017)^15^ regarding Jeffreys1 and the present Section 1 of the Supplementary Material, regarding Jeffreys2). Additionally, both priors generalize easily to the case of meta-regression: the Jeffreys1 prior would coincide with that of Bodnar et al. (2024) for generalized marginal random effects models,^50^ and we derive the Jeffreys2 prior for meta-regression in Section 1 of the Supplementary Material. We do not further consider meta-regression in the main text.

### 3.2 The posterior under each prior

For either prior, since 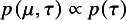 , the marginal posterior on 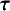 is:^15^


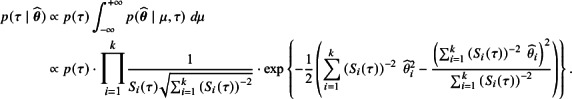

In turn, the conditional posterior of 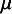 , given 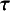 , is normal:^9^ ^,^ ^15^ ^,^ ^21^


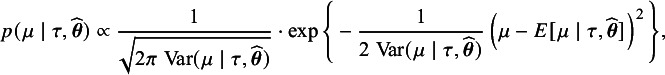

where: 

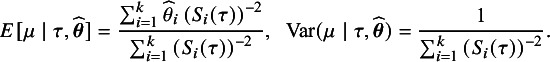

Thus, the joint posterior 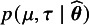 can be decomposed into the two tractable components 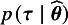 and 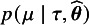 .^9^ Given this observation, Röver and others^9^ ^,^ ^51^ developed theory and software for a discrete approximation to the joint posterior 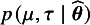 and the marginal posterior on 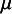 , given by the mixture distribution: 

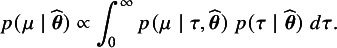

The discrete approximation approach does not require sampling via mixed-chain Monte Carlo (MCMC) and is implemented in the R package bayesmeta.^9^ ^,^ ^51^ We use this package in our simulations and applied example.

With approximations to the joint and marginal posteriors in hand, point estimates can be defined in terms of various measures of central tendency, such as the posterior mode, median, or mean. For either prior, 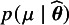 appears to be nearly symmetric in many cases (e.g., Figure 4), so the three measures of central tendency will often agree closely. However, this is not the case for 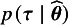 , which is asymmetric under either prior. Existing work on the Jeffreys1 prior focused primarily on posterior means and medians,^15^ but we focus on posterior modes given their aforementioned theoretical advantages.^26^ Indeed, as discussed in Section 4.4, our simulations indicated that posterior modes for 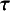 provided substantially lower bias, root mean square error (RMSE), and mean absolute error (MAE) than did posterior means and medians. As in ML estimation, point estimates can be defined either in terms of the marginal or the joint mode. In the Bayesian context, the marginal mode represents the value of a given parameter (e.g., 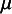 ) that maximizes the posterior for that parameter alone, marginalizing over the other parameter (e.g., 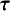 ). In contrast, the joint mode represents the values of both parameters that jointly maximize the joint posterior. We consider marginal modes in this paper to provide a more direct comparison to marginal ML estimation, which is the usual implementation for meta-analysis.

**Figure 2:**
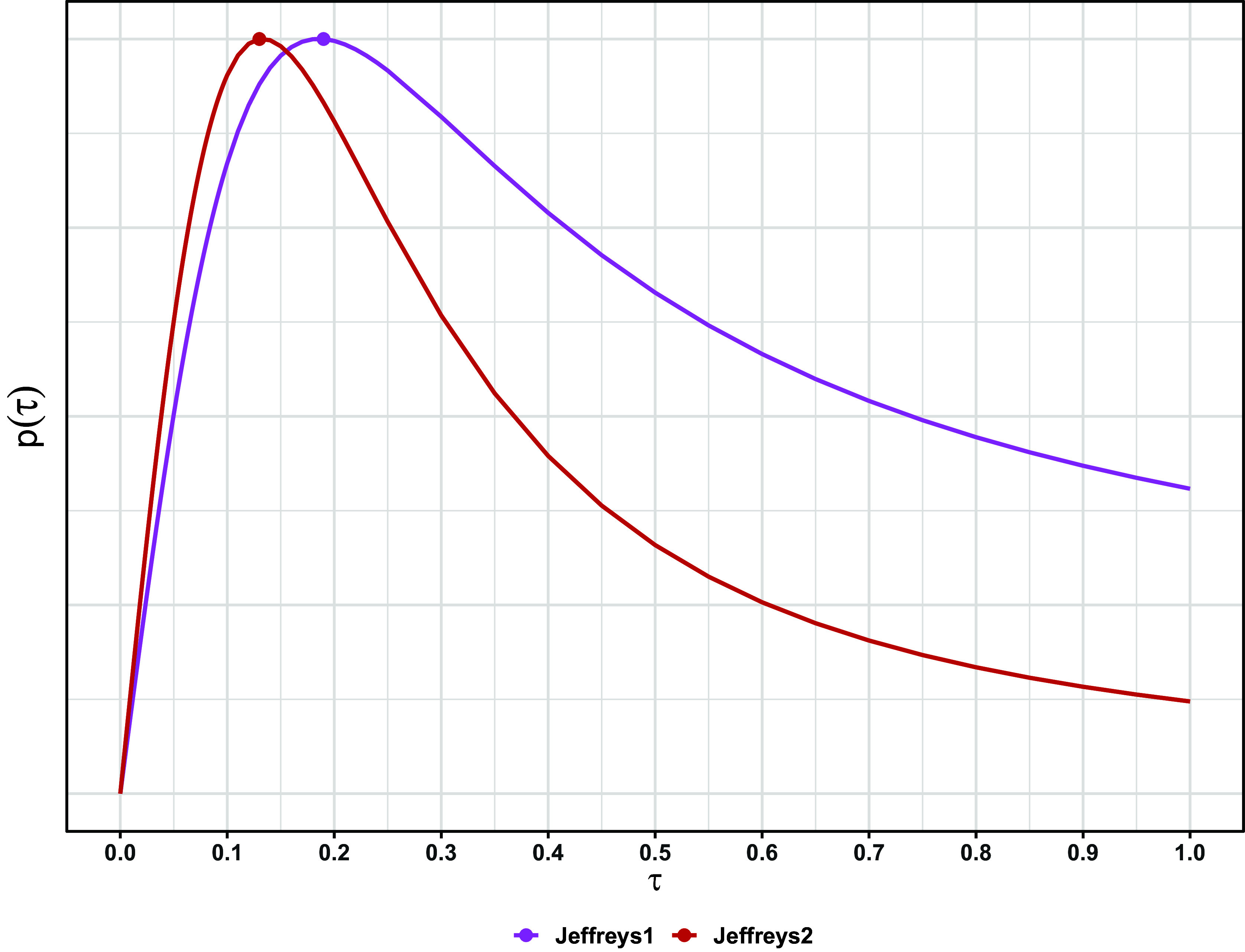
Priors on 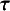 for the meta-analysis on all-cause death ( 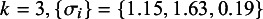 ). Points are maxima. The priors have been scaled to have the same maximum height.

**Figure 3:**
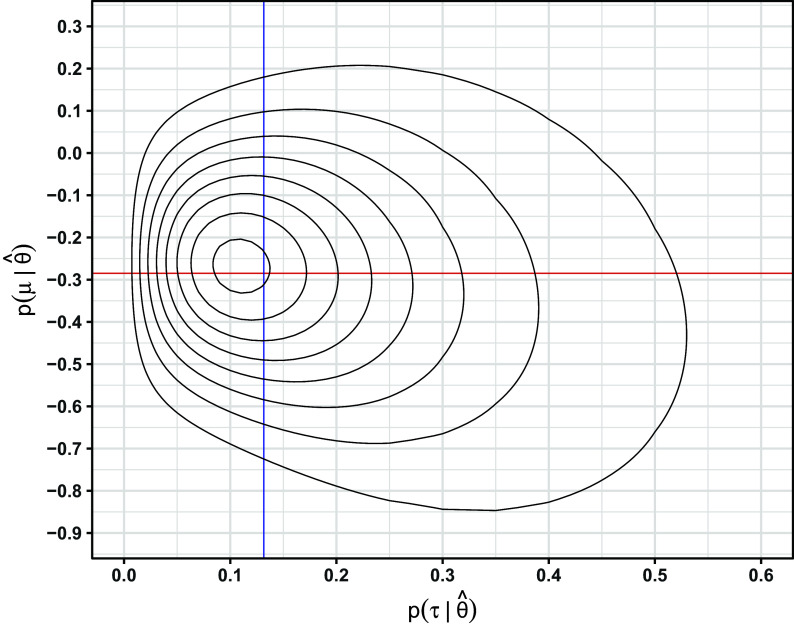
Joint posterior under the Jeffreys2 prior for the meta-analysis on all-cause death ( 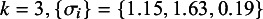 ). Horizontal red line: marginal posterior mode of 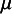 . Vertical blue line: marginal posterior mode of 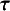 .

**Figure 4:**
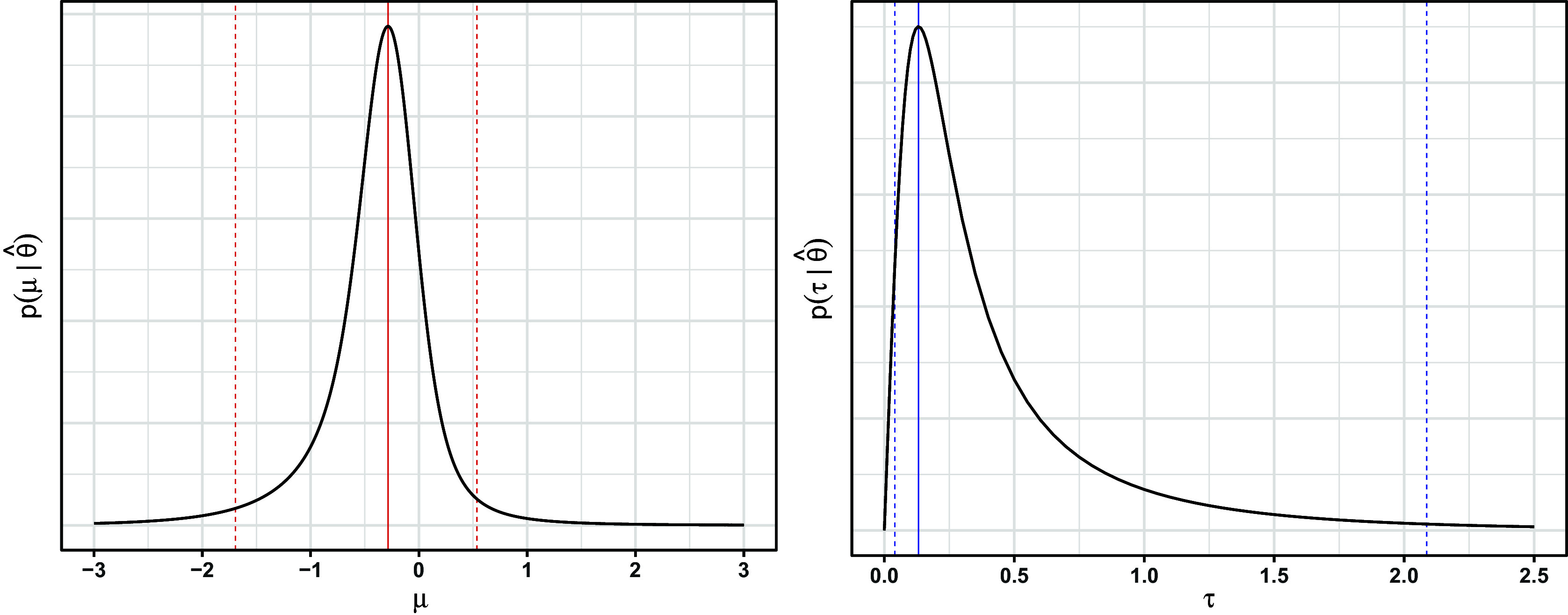
Marginal posteriors under the Jeffreys2 prior for the meta-analysis on all-cause death. Solid vertical lines: marginal posterior modes. Dashed vertical lines: limits of 95% intervals.

**Figure 5:**
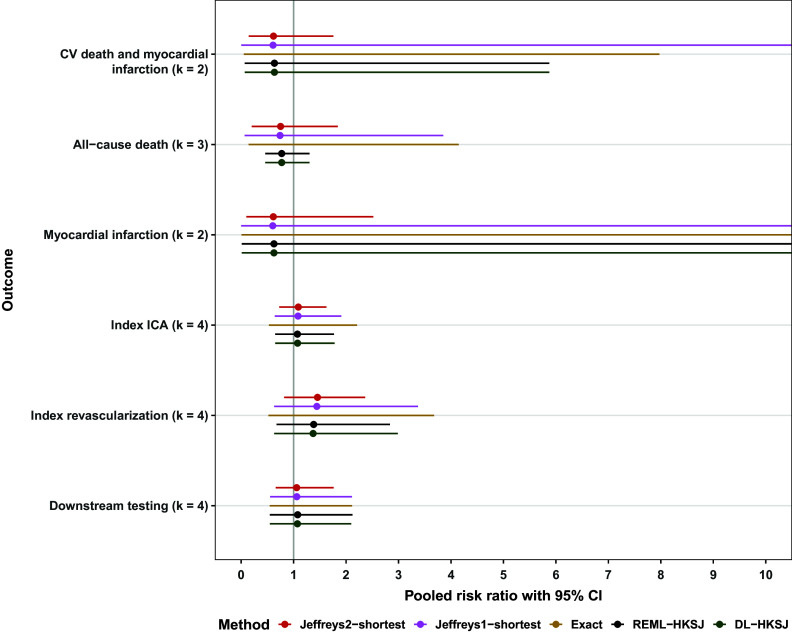
Interval limits greater than 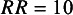 are truncated. The exact method does not yield point estimates. CI: credible interval.

Also analogously to ML estimation, symmetric Wald credible intervals are sometimes constructed for Bayesian estimates by approximating the posterior as asymptotically normal around the posterior mode, with a variance–covariance matrix equal to the inverse of the Hessian of the negative log-posterior evaluated at the posterior mode.^21^ However, just as Wald intervals around the ML estimate can perform poorly if the likelihood is asymmetric, Wald intervals around the posterior mode can likewise perform poorly if the posterior is asymmetric.^52^ To obtain appropriately asymmetric posterior intervals, we consider two approaches. First, a central (also called “equal-tailed”) 95% posterior quantile interval can be obtained by taking the 2.5th and 97.5th quantiles of the estimated posterior distribution. Second, the shortest possible 95% posterior quantile interval can be obtained numerically; this interval is equivalent to a highest posterior density interval for unimodal distributions.^21^ In our simulations and applied example, we obtain both types of intervals from the R package bayesmeta.^9^

### 3.3 Theoretical and substantive distinctions between the priors

The distinction between the Jeffreys1 and Jeffreys2 priors invokes theoretical and substantive considerations that pertain in general to multiparameter Jeffreys priors. Jeffreys and others have argued that multiparameter Jeffreys priors are appropriate if one wishes to estimate all of the parameters (i.e., both 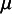 and 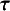 in meta-analysis), but not if one wishes to estimate only a subset of the parameters (i.e., only 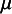 ), with the others treated as nuisance parameters.^24^ ^,^ ^25^ ^,^ ^53^ As noted in the Introduction, a random-effects meta-analysis should generally involve estimation and reporting of 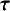 (or related metrics^32^ ^,^ ^54^ ^,^ ^55^) in addition to 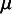 , which suggests consideration of the Jeffreys2 prior. On the other hand, in general location-scale problems, Jeffreys recommended obtaining the prior with respect to only the scale parameters, holding constant the location parameters.^24^ ^,^ ^53^ This would correspond to the Jeffreys1 prior. Jeffreys’ recommendation was motivated by problems that can arise when the number of location parameters increases with the sample size, similarly to the well-known Neyman–Scott problem in which the ML estimator fails to be consistent.^24^ ^,^ ^53^ Interestingly, Firth later showed that in a specific, severe version of the Neyman–Scott problem, the multiparameter Jeffreys prior (i.e., the Firth correction) in fact leads to a consistent and exactly unbiased estimator.^26^ This was unexpected given that the asymptotic arguments justifying the Firth correction are violated with an increasing number of parameters.^26^ Of course, in the present setting of random-effects meta-analysis, the number of parameters is fixed, so this potential issue does not arise in the first place. Our view is that existing substantive and theoretical considerations do not clearly rule out either prior as inappropriate for random-effects meta-analysis, so our simulation study evaluates both.

## 4 Simulation study

We designed the simulation study to closely parallel that of Langan et al. (2019),^7^ which in turn was designed to address many of the limitations of previous simulation studies.^11^ As detailed below, we considered meta-analyses with binary outcomes (with effect sizes on the log-odds ratio scale) and with continuous outcomes (with effect sizes on the Hedges’ *g* scale^56^), with as few as 2 studies, with varying amounts of heterogeneity, with varying means and outcome probabilities (for binary outcomes), and with varying distributions of within-study sample sizes. Because we assessed a variety of parametric, semiparametric, and nonparametric methods, we preliminarily investigated robustness to parametric misspecification by considering exponentially distributed population effects in addition to normally distributed effects.

### 4.1 Point and interval estimation methods

Table 1 lists the methods assessed in our simulation study. We assessed both Jeffreys priors. For point estimation under each prior, we primarily considered marginal posterior modes but secondarily investigated posterior means and medians (Section 2.2 of the Supplementary Material). Regarding interval estimation for 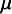 , central and shortest intervals were generally quite similar, so we only show results for shortest intervals. Regarding interval estimation for 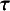 , we consider both types of intervals for each prior, termed “Jeffreys1-shortest,” “Jeffreys1-central,” “Jeffreys2-shortest,” and “Jeffreys2-central.”

**Table 1:** Methods assessed in simulation study

Abbreviation	Method
Point estimation	
ML	Maximum likelihood
REML	Restricted maximum likelihood
DL	Dersimonian–Laird
DL2	Two-step Dersimonian–Laird
PM	Paule–Mandel
Jeffreys1	Marginal posterior mode under Jeffreys1 prior
Jeffreys2	Marginal posterior mode under Jeffreys2 prior
Interval estimation	
HKSJ	Hartung–Knapp–Sidik–Jonkman interval for 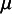 (suffix for moments estimators and likelihood methods)
Qprofile	Q-profile interval for 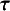 (suffix for moments estimators and likelihood methods)
ML-profile	Maximum likelihood profile interval
Exact	Exact interval for 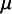
Jeffreys1-shortest, Jeffreys2-shortest	Shortest interval
Jeffreys1-central, Jeffreys2-central	Central (equal-tailed) interval. Results only shown for interval estimation for 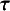 .
Boot-BCa 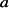	Nonparametric bias-corrected and accelerated bootstrap
Boot-perc 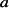	Nonparametic percentile bootstrap

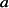
 In pilot tests for the scenarios with 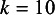 , the bootstrap methods were not competitive with other methods, so these computationally intensive methods were not run for other sample sizes.

We compared the performance of both Jeffreys priors to that of several existing frequentist methods that were described in Section 2. We selected methods that have performed well in existing, large simulation studies or that have desirable theoretical properties, such as providing appropriately asymmetric intervals for 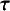 .^6^ ^,^ ^7^ ^,^ ^18^ ^,^ ^39^ ^,^ ^48^ ^,^ ^57^ For point estimation, the comparison methods were ML estimation, REML, DL, DL2, and PM. Regarding interval estimation for 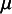 , we considered HKSJ intervals for each frequentist estimation method, ML profile intervals (ML-profile), exact intervals,^18^ nonparametric BCa bootstrap intervals, and nonparametric percentile bootstrap intervals.^45^ ^,^ ^46^ Regarding interval estimation for 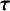 , we considered Q-profile intervals for each frequentist estimation method, as well as ML-profile and both bootstrap intervals. We implemented all frequentist methods and intervals using the R package metafor ^58^ with the following exceptions: we implemented ML-profile using custom R code, the exact method using the R package rma.exact,^18^ and the bootstrap methods using the R package boot.^59^

### 4.2 Data generation

Table 2 summarizes the simulation parameters we manipulated, which were similar to those of Langan et al.’s (2019) simulation study.^7^ We considered continuous outcomes with point estimates on the Hedges’ *g* scale^56^ as well as binary outcomes with point estimates on the log-odds ratio scale. We considered both normally distributed and exponentially distributed population effects; in the latter case, the assumptions for all point estimators except the moments estimators were violated. Statistical theory suggests that all methods would perform comparably for very large meta-analyses with normal effects, and accordingly our focus is on point and interval estimation for smaller meta-analyses ( 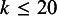 ). Our primary simulations reported in the main text are those with 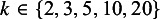 . We additionally ran simulations with 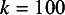 to confirm asymptotic behavior (Section 3 of the Supplementary Material). Because the bootstrap intervals required much more computational time than the other methods, we first pilot-tested them in all scenarios with a single sample size ( 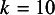 ) to assess whether these methods were competitive with other methods.

**Table 2:** Possible values of simulation parameters

Simulation parameter	Possible values
Outcome type	Continuous (Hedges’ *g*), binary (log-odds ratio)
*k*	2, 3, 5, 10, 20, 100 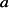
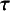	0.01, 0.05, 0.10, 0.20, 0.50
Distribution of population effects	Normal, exponential
Distribution of within-study *N*	All 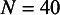 , all 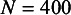 , 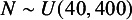 , 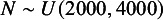
For continuous outcomes only:	
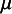	0.5
For binary outcomes only:	
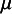	0, 0.5, 1.1, 2.3
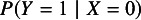	0.05, 0.1, 0.50

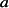
 Results for scenarios with 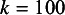 appear in the Supplementary Material; these scenarios are excluded from aggregated results in the main text.

Data generation proceeded as follows. For each simulation iterate, we generated a meta-analysis whose underlying population effects ( 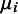 ) were either normal or exponential. Normal population effects were generated as 
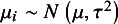
 , where we varied 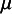 and 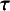 as indicated in Table 2. Exponential population effects were generated from an appropriately scaled and shifted distribution to achieve the desired population moments, 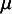 and 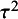 . For each study in the meta-analysis, we generated a total sample size *N* from one of the four distributions listed in Table 2. We then simulated individual participant data, such that 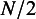 participants were allocated to a treatment group, and the other 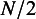 to a control group. In scenarios with a continuous outcome, we simulated outcomes with a mean of 0 in the control group and 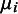 in the treatment group, and with a standard deviation of 1 within each group. We then estimated the standardized mean difference using the Hedges’ *g* correction.^56^ ^,^ ^58^ We used the standard large-sample approximation for studies’ standard errors (Eq. (8) in Hedges (1982)^60^): (5)

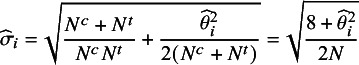

where 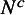 and 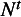 are the within-group sample sizes, which were both equal to 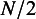 in our simulations. The expectation of this estimator is approximately 
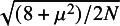
 .

In scenarios with a binary outcome, we simulated outcomes from a logistic model such that: 

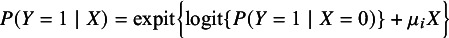

where 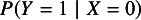 was a scenario parameter that we manipulated among the values listed in Table 2. We then estimated the odds ratio; to handle potential zero cell counts when present, we added 0.5 to each table cell when any cells had a count of zero.^58^

We expected that for binary outcomes and small within-study sample sizes, certain extreme combinations of scenario parameters (e.g., 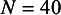 and 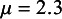 , corresponding to an extreme odds ratio of 10) would result in biased within-study odds ratios.^26^ ^,^ ^61^ In pilot simulations, we identified combinations of scenario parameters that resulted in within-study absolute bias of greater than 0.05. We excluded these combinations of scenario parameters since our focus is on bias arising from meta-analytic estimation methods rather than from within-study bias. After excluding these combinations of simulation parameters, we ultimately simulated 240 unique scenarios for continuous outcomes and 2267 for binary outcomes.

### 4.3 Performance metrics

For each scenario, we assessed the point estimators’ performance and variability in terms of their bias, MAE, and RMSE, defined in the usual frequentist sense. That is, for a generic parameter 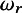 that varies across 500 simulation iterates, *r*: 

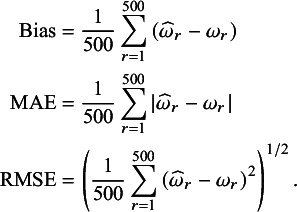

For each scenario, we assessed interval estimation in terms of the frequentist coverage and width of 95% confidence or credible intervals. Some methods’ intervals exhibited over-coverage in some scenarios but exhibited under-coverage in others. Therefore, when aggregating results across scenarios, we also consider the percentage of scenarios in which each method achieved approximately nominal coverage, defined stringently as having coverage 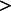 94%. In the Discussion, we expand upon our reasons for assessing frequentist properties of Bayesian methods, and the implications of this approach. We did not assess statistical power. Although *p*-values can certainly be useful when interpreted as continuous measures of evidence, we concur with others’ longstanding concerns about bright-line significance testing,^62^ ^,^ ^63^ a practice that has contributed to striking misinterpretations of published meta-analyses^55^ ^,^ ^64^ and likely also to publication bias.

### 4.4 Results

Given the large number of scenarios, some aggregation is necessary to display the results compactly. In the main text, we provide line plots that stratify by *k*, 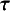 , the distribution of population effects, and the outcome type, and that aggregate over distributions of *N* and, for binary outcomes, over 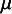 and 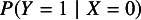 . Because the direction of a given estimator’s bias could differ across scenarios, we depict each estimator’s bias across scenarios using boxplots instead of line plots to avoid any aggregation across scenarios. For the other performance metrics, we additionally provide a series of tables that consider average performances within subsets of scenarios defined by the outcome type and *k* (Tables 3–10). Comprehensive simulation results for each individual scenario are publicly available as a dataset (https://osf.io/9qfah).

**Table 3:** Scenarios with continuous outcomes; 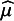 point and interval estimation

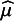 MAE		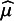 RMSE		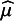 coverage		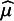 coverage 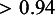		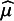 CI width	
All	0.09	All	0.12	Jeffreys1-shortest	0.98	Jeffreys1-shortest	0.89	ML-profile	0.51
				Exact	0.97	Exact	0.84	Jeffreys2-shortest	0.67
				Jeffreys2-shortest	0.96	Jeffreys2-shortest	0.72	DL-HKSJ	1.19
				DL-HKSJ	0.94	DL-HKSJ	0.64	DL2-HKSJ	1.19
				DL2-HKSJ	0.94	DL2-HKSJ	0.64	ML-HKSJ	1.19
				ML-HKSJ	0.94	ML-HKSJ	0.64	PM-HKSJ	1.19
				PM-HKSJ	0.94	PM-HKSJ	0.64	REML-HKSJ	1.19
				REML-HKSJ	0.94	REML-HKSJ	0.64	Exact	1.36
				ML-profile	0.93	ML-profile	0.50	Jeffreys1-shortest	1.57

*Note*: Methods are sorted from best to worst performance within each column (or alphabetically for ties); coverage is sorted from highest to lowest. MAE: Mean absolute error. RMSE: Root mean square error. CI: 95% confidence or credible interval. Coverage 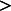 0.94: Percent of scenarios for which coverage probability was at least 0.94. “All”: All methods performed equally to two decimal places.

**Table 4:** Scenarios with continuous outcomes; 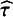 point and interval estimation

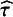 MAE		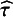 RMSE		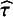 coverage		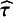 coverage 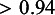		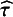 CI width	
Jeffreys1	0.09	Jeffreys2	0.10	DL-Qprofile	0.93	Jeffreys1-shortest	0.74	ML-profile	0.42
Jeffreys2	0.09	Jeffreys1	0.11	DL2-Qprofile	0.93	Jeffreys2-shortest	0.62	Jeffreys2-shortest	0.50
ML	0.09	ML	0.11	Jeffreys1-shortest	0.93	ML-profile	0.62	Jeffreys2-central	0.62
DL	0.10	DL	0.12	ML-Qprofile	0.93	Jeffreys2-central	0.58	Jeffreys1-shortest	1.37
DL2	0.10	DL2	0.12	PM-Qprofile	0.93	DL-Qprofile	0.56	DL-Qprofile	1.47
PM	0.10	PM	0.12	REML-Qprofile	0.93	DL2-Qprofile	0.56	DL2-Qprofile	1.47
REML	0.10	REML	0.12	ML-profile	0.92	Jeffreys1-central	0.56	ML-Qprofile	1.47
				Jeffreys2-shortest	0.91	ML-Qprofile	0.56	PM-Qprofile	1.47
				Jeffreys2-central	0.79	PM-Qprofile	0.56	REML-Qprofile	1.47
				Jeffreys1-central	0.77	REML-Qprofile	0.56	Jeffreys1-central	2.40

*Note*: Methods are sorted from best to worst performance within each column (or alphabetically for ties); coverage is sorted from highest to lowest. MAE: Mean absolute error. RMSE: Root mean square error. CI: 95% confidence or credible interval. Coverage 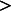 0.94: Percent of scenarios for which coverage probability was at least 0.94.

**Table 5:** Scenarios with binary outcomes; 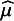 point and interval estimation

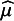 MAE		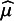 RMSE		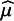 coverage		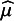 coverage 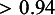		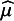 CI width	
All	0.16	All	0.20	Jeffreys1-shortest	0.99	Jeffreys1-shortest	0.95	ML-profile	0.96
				Exact	0.98	Exact	0.93	Jeffreys2-shortest	1.38
				Jeffreys2-shortest	0.98	Jeffreys2-shortest	0.89	ML-HKSJ	2.05
				DL-HKSJ	0.95	DL-HKSJ	0.73	REML-HKSJ	2.07
				DL2-HKSJ	0.95	DL2-HKSJ	0.73	DL-HKSJ	2.08
				ML-HKSJ	0.95	ML-HKSJ	0.73	DL2-HKSJ	2.08
				ML-profile	0.95	ML-profile	0.73	PM-HKSJ	2.08
				PM-HKSJ	0.95	PM-HKSJ	0.73	Exact	2.64
				REML-HKSJ	0.95	REML-HKSJ	0.73	Jeffreys1-shortest	3.26

*Note*: Methods are sorted from best to worst performance within each column (or alphabetically for ties); coverage is sorted from highest to lowest. MAE: Mean absolute error. RMSE: Root mean square error. CI: 95% confidence or credible interval. Coverage 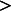 0.94: Percent of scenarios for which coverage probability was at least 0.94. “All”: All methods performed equally to two decimal places.

**Table 6:** Scenarios with binary outcomes; 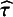 point and interval estimation

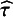 MAE		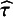 RMSE		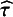 coverage		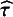 coverage 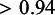		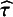 CI width	
ML	0.13	ML	0.16	ML-profile	0.96	ML-profile	0.82	ML-profile	0.80
DL	0.15	Jeffreys2	0.17	DL-Qprofile	0.94	Jeffreys1-shortest	0.78	Jeffreys2-shortest	1.02
DL2	0.15	DL	0.20	DL2-Qprofile	0.94	Jeffreys2-shortest	0.72	Jeffreys2-central	1.28
PM	0.15	DL2	0.20	ML-Qprofile	0.94	DL-Qprofile	0.71	DL2-Qprofile	2.17
REML	0.15	Jeffreys1	0.20	PM-Qprofile	0.94	ML-Qprofile	0.71	DL-Qprofile	2.19
Jeffreys2	0.16	PM	0.20	REML-Qprofile	0.94	PM-Qprofile	0.71	ML-Qprofile	2.19
Jeffreys1	0.18	REML	0.20	Jeffreys1-shortest	0.90	REML-Qprofile	0.71	PM-Qprofile	2.19
				Jeffreys2-shortest	0.89	DL2-Qprofile	0.70	REML-Qprofile	2.19
				Jeffreys2-central	0.68	Jeffreys2-central	0.51	Jeffreys1-shortest	2.86
				Jeffreys1-central	0.64	Jeffreys1-central	0.45	Jeffreys1-central	5.06

*Note*: Methods are sorted from best to worst performance within each column (or alphabetically for ties); coverage is sorted from highest to lowest. MAE: Mean absolute error. RMSE: Root mean square error. CI: 95% confidence or credible interval. Coverage 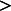 0.94: Percent of scenarios for which coverage probability was at least 0.94.

**Table 7:** Scenarios with continuous outcomes, 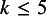 ; 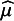 point and interval estimation

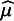 MAE		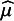 RMSE		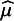 coverage		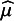 coverage 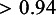		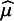 CI width	
All	0.12	All	0.15	Jeffreys1-shortest	0.99	Jeffreys1-shortest	0.93	ML-profile	0.66
				Exact	0.98	Exact	0.91	Jeffreys2-shortest	0.92
				Jeffreys2-shortest	0.96	Jeffreys2-shortest	0.76	ML-HKSJ	1.78
				DL-HKSJ	0.94	DL-HKSJ	0.68	DL-HKSJ	1.79
				DL2-HKSJ	0.94	DL2-HKSJ	0.68	DL2-HKSJ	1.79
				ML-HKSJ	0.94	ML-HKSJ	0.68	PM-HKSJ	1.79
				PM-HKSJ	0.94	PM-HKSJ	0.68	REML-HKSJ	1.79
				REML-HKSJ	0.94	REML-HKSJ	0.68	Exact	2.06
				ML-profile	0.92	ML-profile	0.52	Jeffreys1-shortest	2.40

*Note*: Methods are sorted from best to worst performance within each column (or alphabetically for ties); coverage is sorted from highest to lowest. MAE: Mean absolute error. RMSE: Root mean square error. CI: 95% confidence or credible interval. Coverage 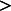 0.94: Percent of scenarios for which coverage probability was at least 0.94. “All”: All methods performed equally to two decimal places.

**Table 8:** Scenarios with continuous outcomes, 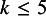 ; 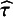 point and interval estimation

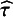 MAE		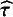 RMSE		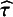 coverage		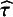 coverage 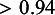		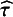 CI width	
Jeffreys2	0.10	Jeffreys2	0.12	Jeffreys1-shortest	0.97	Jeffreys1-shortest	0.87	ML-profile	0.54
Jeffreys1	0.11	Jeffreys1	0.13	DL-Qprofile	0.94	Jeffreys2-shortest	0.72	Jeffreys2-shortest	0.66
ML	0.11	ML	0.13	DL2-Qprofile	0.94	ML-profile	0.68	Jeffreys2-central	0.85
DL	0.12	DL	0.15	Jeffreys2-shortest	0.94	Jeffreys2-central	0.65	Jeffreys1-shortest	2.10
DL2	0.12	DL2	0.15	ML-Qprofile	0.94	Jeffreys1-central	0.60	DL2-Qprofile	2.26
PM	0.12	PM	0.15	PM-Qprofile	0.94	DL-Qprofile	0.56	DL-Qprofile	2.27
REML	0.12	REML	0.15	REML-Qprofile	0.94	DL2-Qprofile	0.56	ML-Qprofile	2.27
				ML-profile	0.92	ML-Qprofile	0.56	PM-Qprofile	2.27
				Jeffreys2-central	0.78	PM-Qprofile	0.56	REML-Qprofile	2.27
				Jeffreys1-central	0.76	REML-Qprofile	0.56	Jeffreys1-central	3.80

*Note*: Methods are sorted from best to worst performance within each column (or alphabetically for ties); coverage is sorted from highest to lowest. MAE: Mean absolute error. RMSE: Root mean square error. CI: 95% confidence or credible interval. Coverage 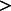 0.94: Percent of scenarios for which coverage probability was at least 0.94.

**Table 9:** Scenarios with binary outcomes, 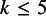 ; 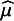 point and interval estimation

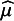 MAE		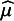 RMSE		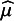 coverage		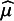 coverage 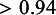		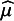 CI width	
All	0.20	All	0.25	Jeffreys1-shortest	1.00	Jeffreys1-shortest	0.98	ML-profile	1.27
				Exact	0.99	Exact	0.97	Jeffreys2-shortest	1.93
				Jeffreys2-shortest	0.99	Jeffreys2-shortest	0.92	ML-HKSJ	3.08
				DL-HKSJ	0.95	ML-profile	0.75	DL-HKSJ	3.13
				DL2-HKSJ	0.95	DL-HKSJ	0.74	DL2-HKSJ	3.13
				ML-HKSJ	0.95	DL2-HKSJ	0.74	PM-HKSJ	3.13
				ML-profile	0.95	ML-HKSJ	0.74	REML-HKSJ	3.13
				PM-HKSJ	0.95	PM-HKSJ	0.74	Exact	4.04
				REML-HKSJ	0.95	REML-HKSJ	0.74	Jeffreys1-shortest	5.05

*Note*: Methods are sorted from best to worst performance within each column (or alphabetically for ties); coverage is sorted from highest to lowest. MAE: Mean absolute error. RMSE: Root mean square error. CI: 95% confidence or credible interval. Coverage 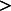 0.94: Percent of scenarios for which coverage probability was at least 0.94. “All”: All methods performed equally to two decimal places.

**Table 10:** Scenarios with binary outcomes, 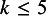 ; 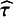 point and interval estimation

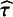 MAE		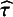 RMSE		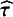 coverage		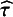 coverage 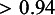		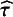 CI width	
ML	0.14	ML	0.18	ML-profile	0.97	Jeffreys1-shortest	0.89	ML-profile	1.04
DL	0.18	Jeffreys2	0.20	DL-Qprofile	0.95	ML-profile	0.85	Jeffreys2-shortest	1.38
DL2	0.18	DL	0.24	DL2-Qprofile	0.95	Jeffreys2-shortest	0.81	Jeffreys2-central	1.80
Jeffreys2	0.18	DL2	0.24	Jeffreys1-shortest	0.95	DL-Qprofile	0.73	DL2-Qprofile	3.30
PM	0.18	REML	0.24	ML-Qprofile	0.95	ML-Qprofile	0.73	DL-Qprofile	3.32
REML	0.18	Jeffreys1	0.25	PM-Qprofile	0.95	PM-Qprofile	0.73	ML-Qprofile	3.32
Jeffreys1	0.22	PM	0.25	REML-Qprofile	0.95	REML-Qprofile	0.73	PM-Qprofile	3.32
				Jeffreys2-shortest	0.93	DL2-Qprofile	0.72	REML-Qprofile	3.32
				Jeffreys2-central	0.65	Jeffreys2-central	0.52	Jeffreys1-shortest	4.43
				Jeffreys1-central	0.59	Jeffreys1-central	0.43	Jeffreys1-central	8.08

*Note*: Methods are sorted from best to worst performance within each column (or alphabetically for ties); coverage is sorted from highest to lowest. MAE: Mean absolute error. RMSE: Root mean square error. CI: 95% confidence or credible interval. Coverage 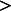 0.94: Percent of scenarios for which coverage probability was at least 0.94.

As described above, our focus is on small meta-analyses. Thus, except where otherwise noted, all subsequent results pertain to scenarios with 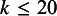 , and we refer to these as “all scenarios.” Although tables and figures show results for both normal and exponential population effects, our prose descriptions focus primarily on scenarios with normal effects; in these scenarios, all methods were correctly specified. We do secondarily discuss how results changed for exponentially distributed effects. Note that figures stratify on effect distribution, while tables aggregate over normal and exponential effects due to space constraints.

#### 4.4.1 Convergence metrics

Other than the exact method and the BCa bootstrap, all methods’ algorithms converged (in the sense of yielding point estimates and/or intervals for 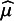 and 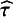 ) in 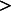 99% of simulated datasets. The exact method is designed only to provide an interval for 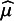 , and its algorithm did so in 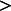 98% of simulated datasets. In the subset of scenarios we ran with the bootstrap methods (i.e., scenarios with 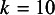 ), the BCa bootstrap only provided an interval for 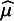 and 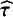 in 67% of datasets. When no interval was provided, this was because the estimated bias correction was infinite, which can happen when empirical influence values are close to zero due to outliers or small sample sizes.

#### Point and interval estimation for 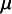


4.4.2

**Figure 6:**
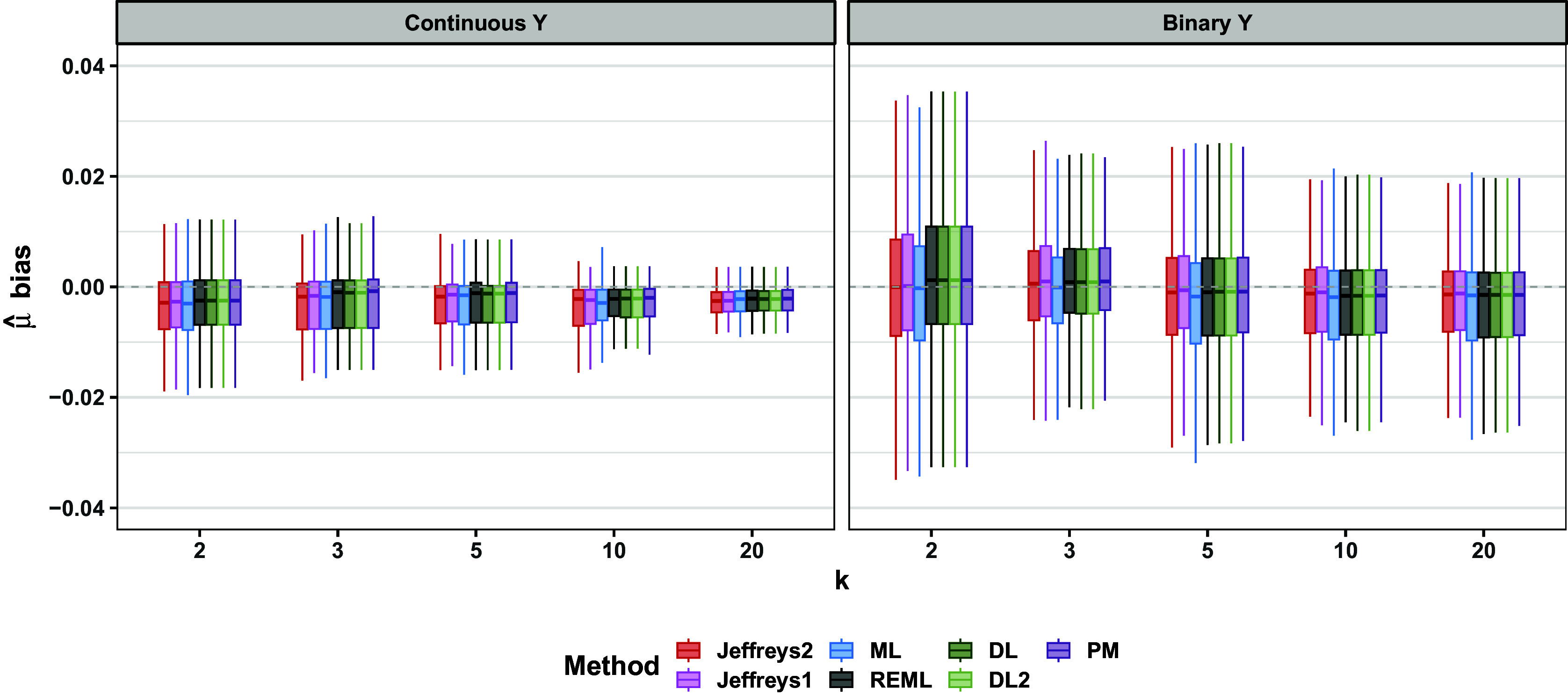
Bias of 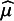 ; all scenarios. Hinges of each boxplot are the 25th, 50th, and 75th percentiles. The upper and lower whiskers extend from the hinge to the minimum or maximum value that is no more than 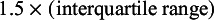 from the nearest hinge.

Consistent with previously published simulations,^10^ all methods performed very similarly for point estimation of 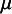 and were approximately unbiased (Figure 6 and Section 2.1 of the Supplementary Material). Across all scenarios, the maximum within-scenario absolute differences between any two methods in bias, RMSE, and MAE respectively were only 0.056, 0.064, and 0.036. Given these relatively minor differences in point estimation for 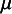 , we primarily discuss interval estimation for this estimand. In pilot tests for 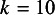 scenarios, the bootstrap methods were not competitive with other methods (Sections 3.7 and 3.8 of the Supplementary Material). Therefore, we did not run these computationally intensive methods 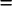 for other sample sizes, and the bootstrap methods are omitted from results in the main text.

**Figure 7:**
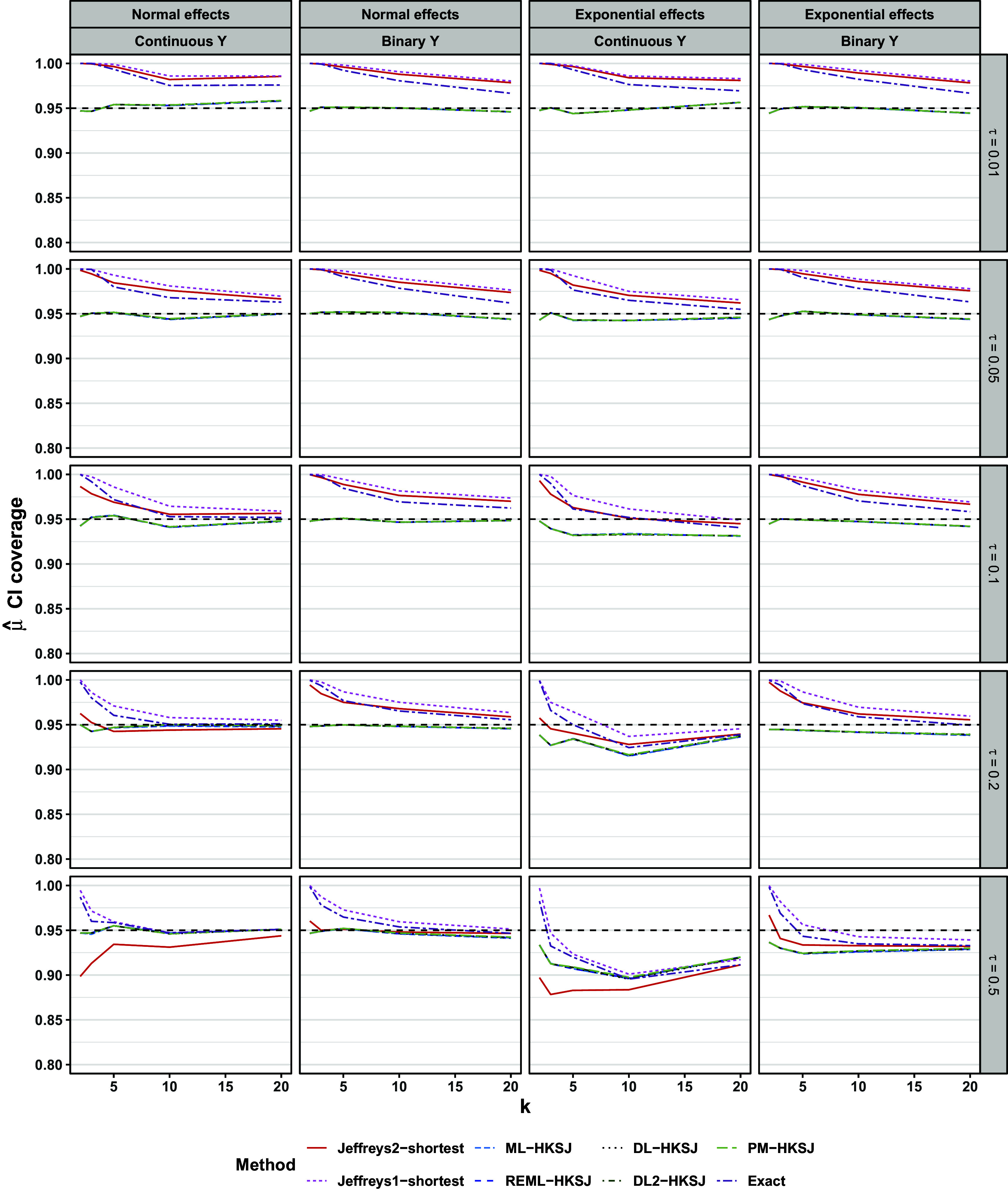
Coverage of CI for 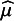 . Lines are slightly staggered horizontally for visibility. Lines are mean performances across scenarios, conditional on *k*, 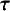 , the distribution of population effects, and the outcome type. All HKSJ methods performed very similarly, so their overlapping lines look like a single grey line.

Figure 7 shows the coverage of 95% intervals. All frequentist methods with HKSJ intervals performed similarly to one another. In scenarios with normal population effects, these methods’ performances were minimally affected by *k* and 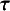 , and coverage was 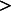 94% in 80% of scenarios. This is a somewhat pessimistic portrayal because coverages for these methods were also rarely below 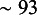 %. ML-profile had coverage 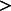 94% in 71% of scenarios with normal effects, but unlike the HKSJ methods, the coverage of ML-profile varied substantially across scenarios. In particular, this method had close to nominal coverage for intermediate levels of heterogeneity and for 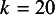 , but exhibited under-coverage for higher heterogeneity values (e.g., 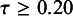 ). The exact interval exhibited over-coverage for smaller values of *k* and close to nominal coverage for 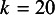 . All of these findings are consistent with previous simulation studies.^10^ ^,^ ^18^

Jeffreys1-shortest and Jeffreys2-shortest intervals had coverage 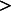 94% in 98% and in 88% of scenarios with normal population effects, respectively. This exceeded the 80% and 71% seen for HKSJ intervals and ML-profile intervals, respectively. In individual scenarios, Jeffreys1-shortest and Jeffreys2-shortest intervals both typically exhibited over-coverage or nominal coverage with one exception: Jeffreys2-shortest intervals exhibited mild under-coverage ( 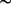 89–93%) for very small meta-analyses ( 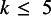 ) that also had a continuous outcome and high heterogeneity ( 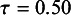 ).

**Figure 8:**
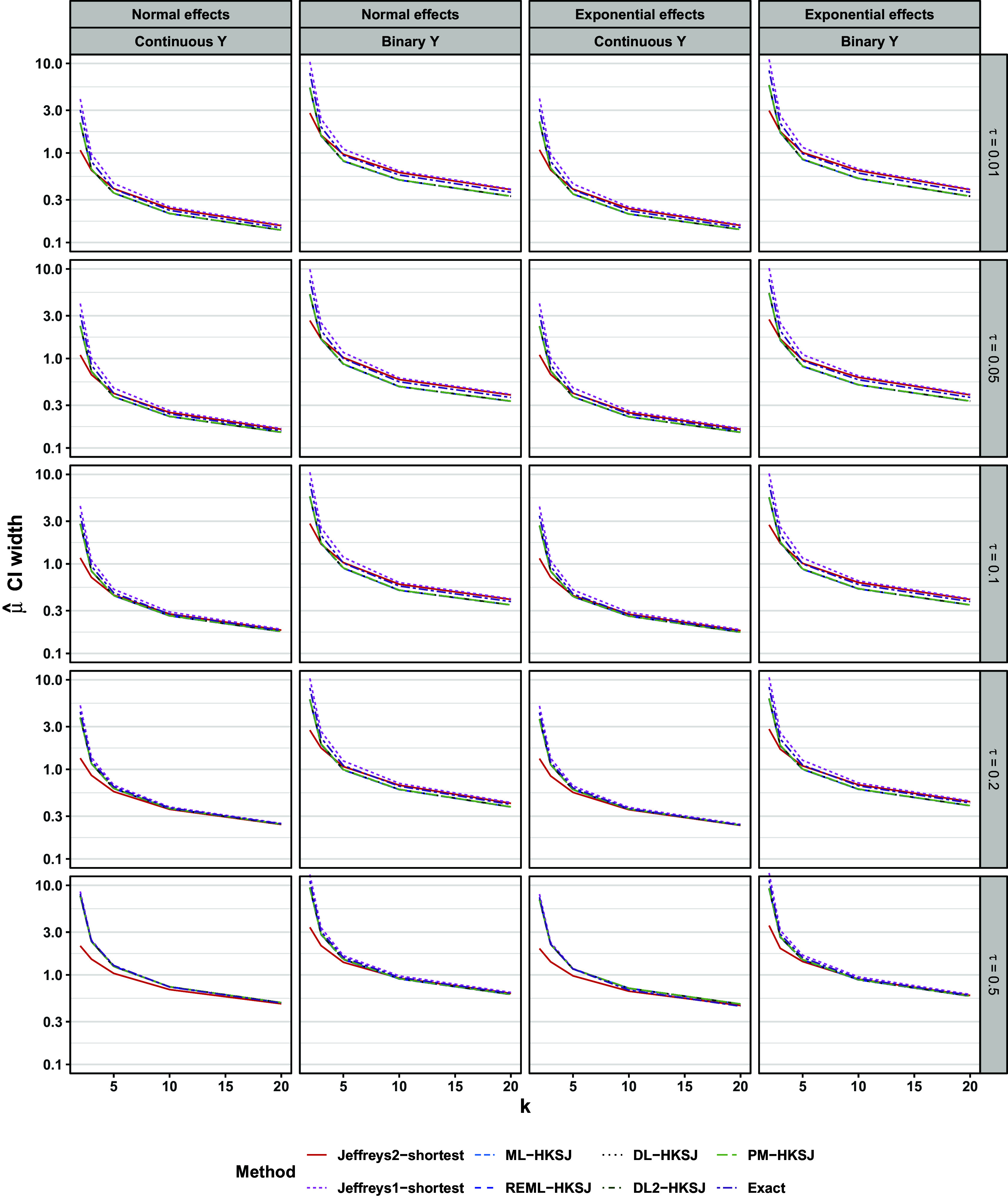
Width of CI for 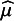 . Lines are slightly staggered horizontally for visibility. Lines are mean performances across scenarios, conditional on *k*, 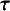 , the distribution of population effects, and the outcome type. Y-axis is on log scale.

Figure 8 shows the width of 95% intervals. For 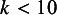 , the different intervals’ widths varied, sometimes substantially. In these scenarios, the ML-profile interval was consistently the narrowest, and was often considerably so for very small meta-analyses. The Jeffreys1-shortest interval was typically the widest of all, especially for very small meta-analyses. On the other hand, the Jeffreys2-shortest interval was typically the second-narrowest after ML-profile, and was substantially narrower than all HKSJ intervals for very small meta-analyses. It may be counterintuitive that the Jeffreys2-shortest interval was narrower than the HKSJ intervals while more consistently achieving at least nominal coverage; we explain this finding in Section 4.4.3 below. For 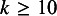 and continuous outcomes, all types of intervals had nearly identical widths. For 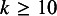 and binary outcomes, both Jeffreys intervals and the exact interval were slightly wider than those of the HKSJ methods, although this should be interpreted in light of the frequentist methods’ slight under-coverage in these scenarios (Figure 7).

In scenarios with exponential population effects, all methods’ relative performances were similar, although coverages declined somewhat when heterogeneity was high ( 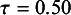 ). This, too, is consistent with previous simulation studies.^10^ Section 3 of the Supplementary Material provides additional results stratified by outcome type. First, results are shown for scenarios with 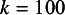 , since these scenarios are excluded from all results in the main text. In those scenarios, as expected from theory, all point estimates performed very similarly regardless of outcome type. For binary outcomes, most methods’ coverage probabilities declined somewhat at 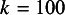 . This finding is consistent with previous simulation results involving rare binary outcomes (Langan et al. (2019)^7^; Appendix Figure 4) and likely reflects known two sources of misspecification when meta-analyzing log-odds ratios. In particular: (1) estimated log-odds ratios are correlated with their estimated standard errors; and (2) the conventional variance estimate is an imperfect approximation especially when there are zero cell counts, a problem that occurs even when adding positive constants to each cell.^65^ ^,^ ^66^ We return to these issues in the Discussion. In these scenarios, Jeffreys methods retained closer to nominal coverage than did the frequentist methods. Additional supplementary tables stratify the results in the main text (i.e., scenarios with 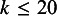 ) into those with fixed versus varying *N* across studies. In all of these strata, the relative rankings of methods’ performances were quite similar to those in the aggregate analyses.

#### Discussion of results regarding 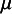


4.4.3

For small meta-analyses ( 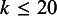 ) with binary outcomes, Jeffreys2-shortest may be a useful method since its intervals had at least nominal coverage (with normal effects) and yet were often considerably narrower than all others except ML-profile, whose coverage was inconsistent across scenarios. To illustrate, we provide some numerical comparisons between Jeffreys2-shortest and REML-HKSJ intervals for meta-analyses of binary outcomes. We compare to a single type of frequentist interval for simplicity. In scenarios with binary outcomes and normal population effects, Jeffreys2-shortest had coverage 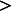 94% in 90% of scenarios, whereas REML-HKSJ did so in 80% of scenarios. Accordingly, Jeffreys2-shortest had coverage at least equal to that of REML-HKSJ in 85% of scenarios. At the same time, the Jeffreys2-shortest interval was on average 27% narrower than the REML-HKSJ interval; and in meta-analyses with 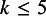 , this efficiency improvement increased to 51%. For binary outcomes, Jeffreys1-shortest did not appear to have clear advantages over Jeffreys2-shortest or the other methods, since the Jeffreys1-shortest interval was wider than even that of the exact method.

For small meta-analyses with continuous outcomes, more caution is warranted when using Jeffreys2-shortest intervals, since they exhibit mild under-coverage ( 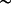 89–93%) for very small meta-analyses ( 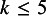 ) that also had high heterogeneity. Since Jeffreys2-shortest provided only modest improvements in efficiency for meta-analyses of continuous outcomes once 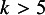 , it may be preferable to conservatively use a frequentist method with an HKSJ interval for continuous outcomes, regardless of *k*. Although the Jeffreys1-shortest interval did, in general, retain at least nominal coverage for continuous outcomes, this interval was again wider than the exact interval, and was considerably wider than the HKSJ intervals.

As noted above, it may be counterintuitive that the Jeffreys2-shortest interval was typically narrower than the HKSJ intervals while more consistently achieving at least nominal coverage. There are two reasons for this finding. First, whereas HKSJ intervals for 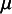 are always symmetric on the analyzed effect scale (i.e., Hedges’ *g* for continuous outcomes and log-odds ratio for binary outcomes), the Jeffreys1-shortest and Jeffreys2-shortest intervals can be symmetric or asymmetric depending on the shape of the posterior (Section 2.3 of the Supplementary Material). Second, within a given scenario, the width of the Jeffreys2-shortest interval was typically much less variable across repeated samples than the HKSJ intervals. Thus, in many scenarios in which the Jeffreys2-shortest interval exhibited over-coverage but comparison methods exhibited nominal or less than nominal coverage, this was because the HKSJ methods often yielded extremely wide intervals under repeated sampling, whereas the Jeffreys2-shortest intervals were bounded within a narrower range (Section 2.3 of the Supplementary Material).

#### Point and interval estimation for 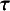


4.4.4

**Figure 9:**
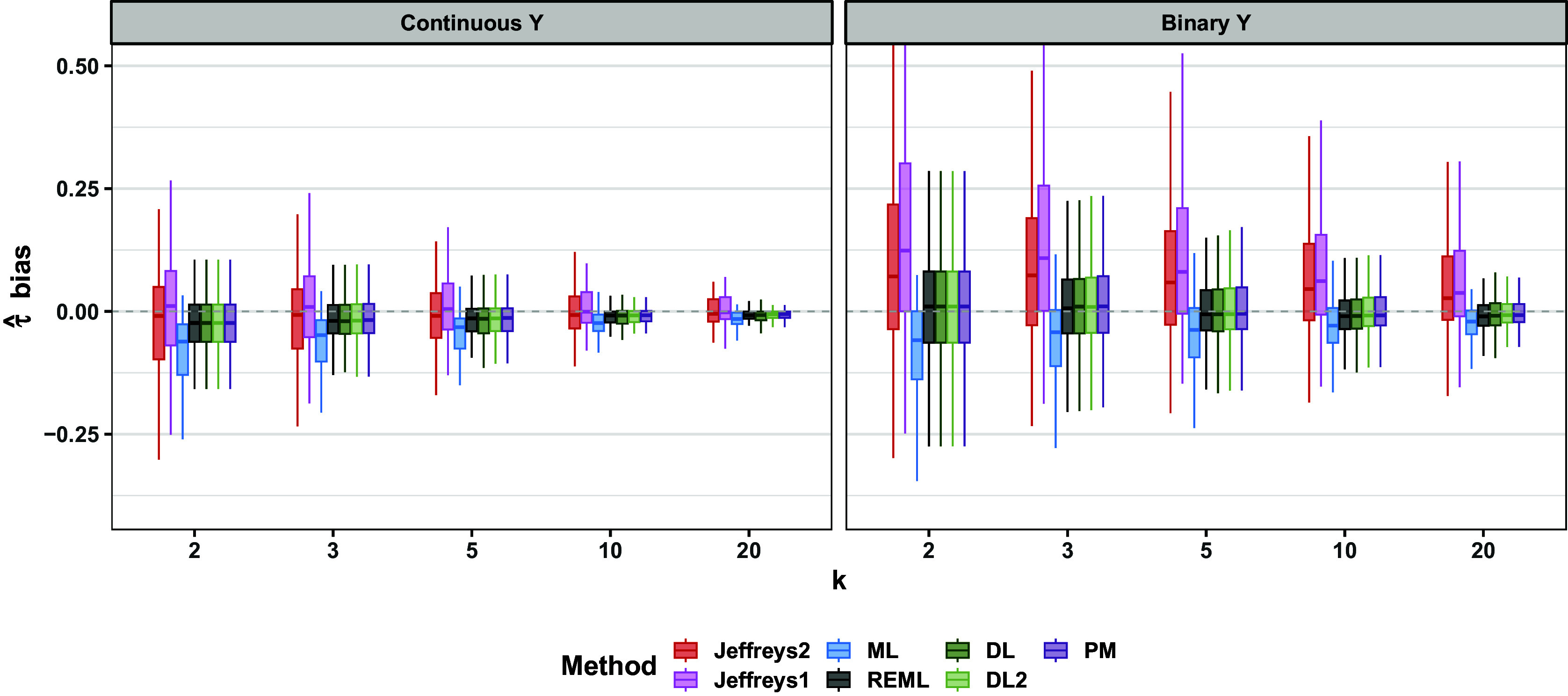
Bias of 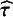 ; all scenarios. Hinges of each boxplot are the 25th, 50th, and 75th percentiles. The upper and lower whiskers extend from the hinge to the minimum or maximum value that is no more than 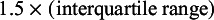 from the nearest hinge.

**Figure 10:**
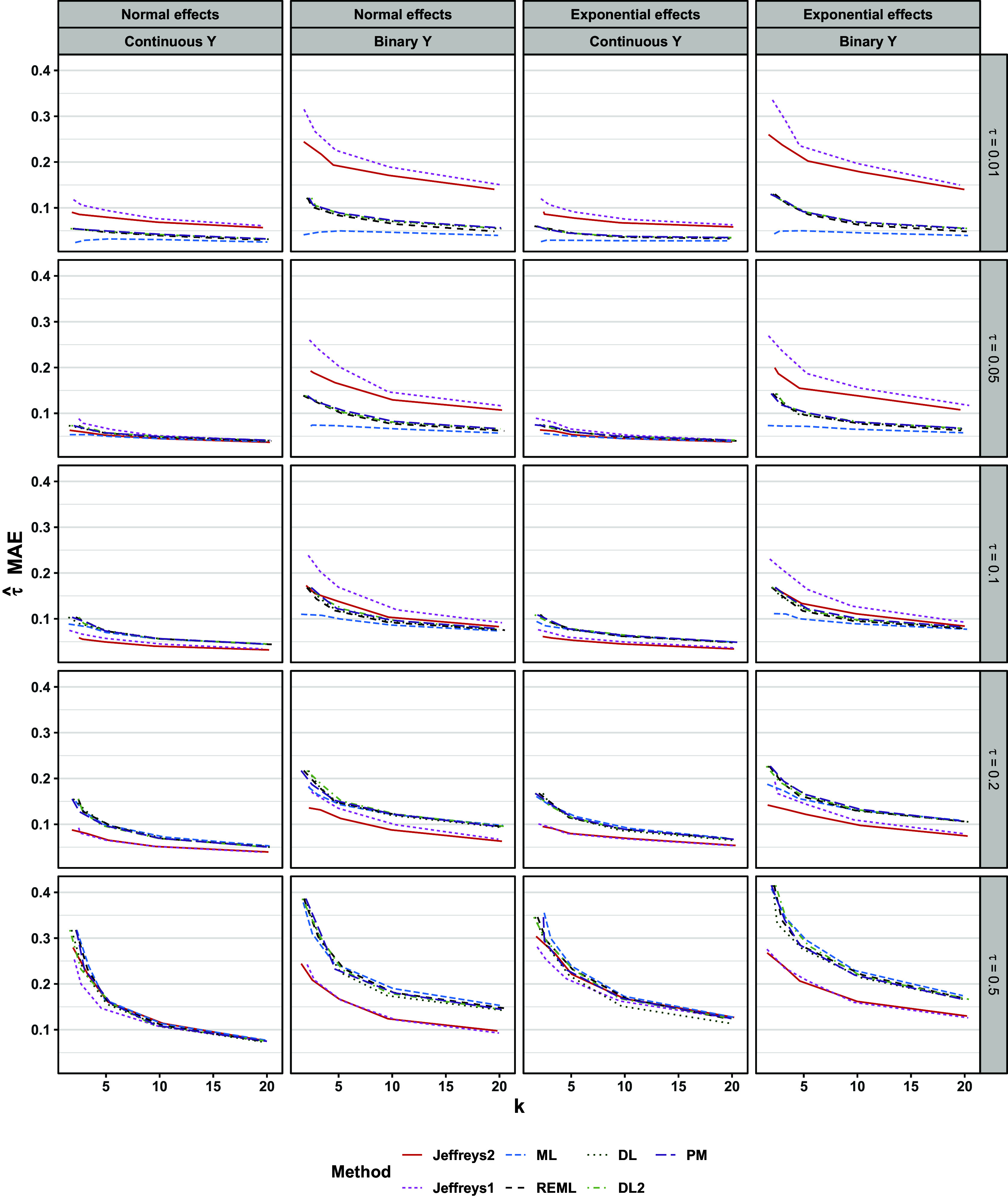
MAE of 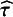 . Lines are slightly staggered horizontally for visibility. Lines are mean performances across scenarios, conditional on *k*, 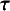 , the distribution of population effects, and the outcome type.

**Figure 11:**
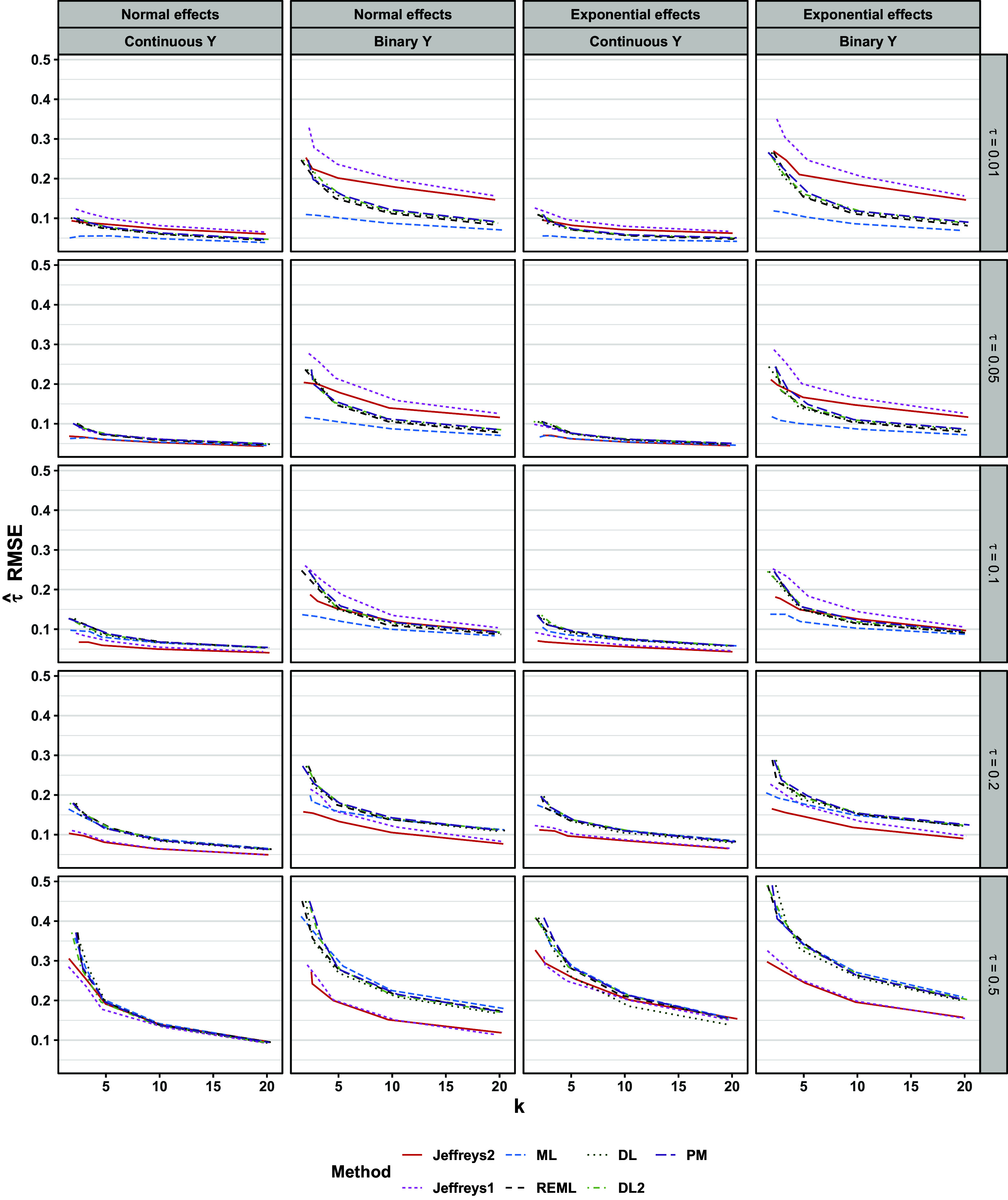
RMSE of 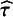 . Lines are slightly staggered horizontally for visibility. Lines are mean performances across scenarios, conditional on *k*, 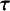 , the distribution of population effects, and the outcome type.

For both continuous and binary outcomes, results for point and interval estimation depended on whether 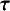 was near the boundary value of zero, especially for the Jeffreys methods. Regarding point estimation, the frequentist methods, especially ML, typically showed a slight negative bias (Figure 9). Point estimates from Jeffreys1 and Jeffreys2 varied more in the sign and magnitude of bias than did the frequentist point estimates (Figure 9). Regarding MAE and RMSE, the frequentist methods DL, DL2, REML, and PM were comparable to one another. In contrast, ML often performed slightly better on these metrics (Figures 10 and 11). Jeffreys1 and Jeffreys2 had comparable MAE and RMSE to one another. Relative to the frequentist methods, Jeffreys1 and Jeffreys2 typically showed comparable MAE and RMSE at midrange values of 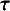 (e.g., 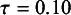 ), showed better MAE and RMSE for 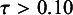 , and showed worse MAE and RMSE for 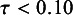 . These patterns were more pronounced for binary outcomes, though the relative rankings of methods were similar for both outcome types. The patterns were similar for both normal and exponential population effects.

**Figure 12:**
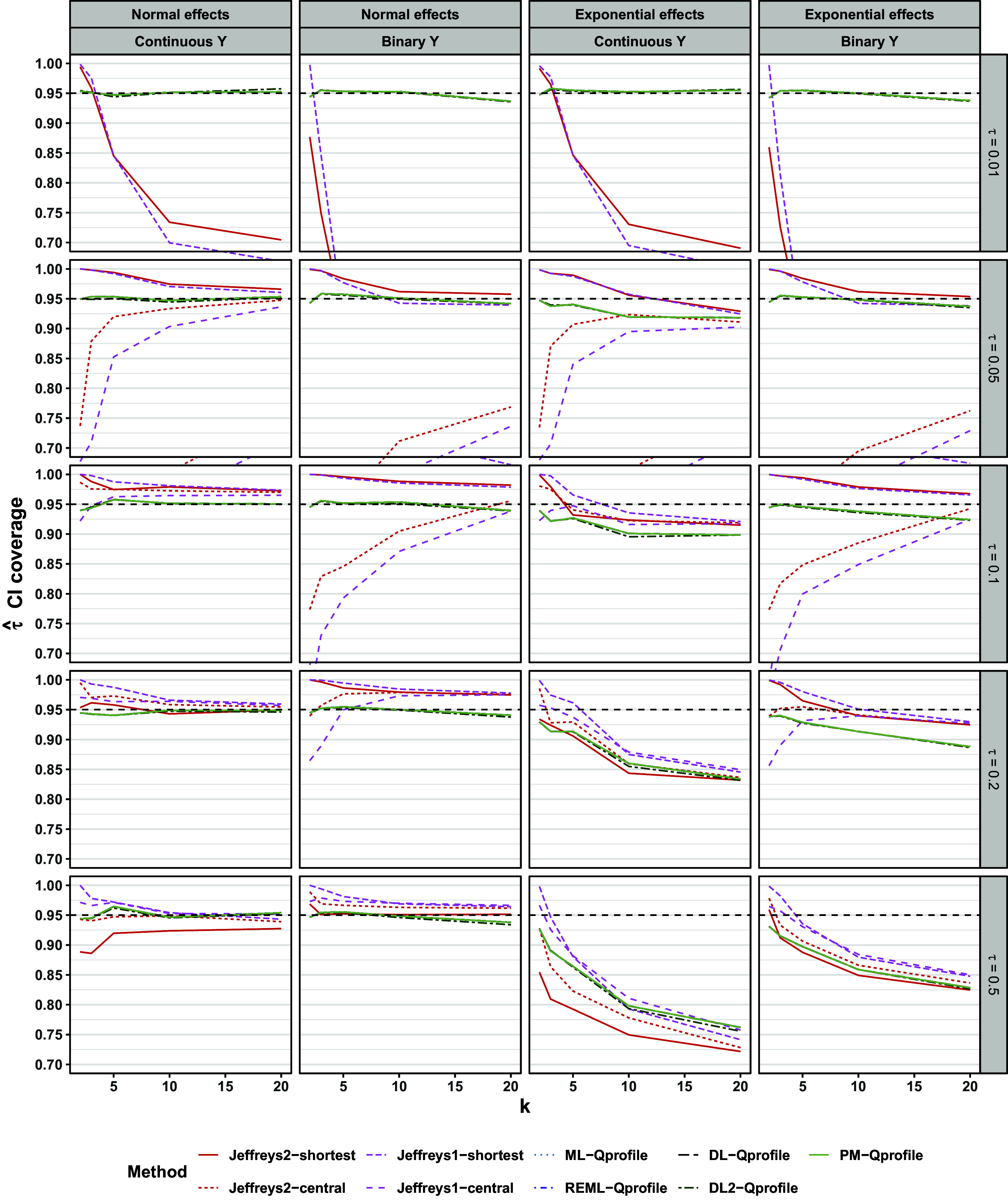
Coverage of CI for 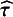 . Lines are slightly staggered horizontally for visibility. Lines are mean performances across scenarios, conditional on *k*, 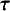 , the distribution of population effects, and the outcome type.

Regarding interval estimation, pilot tests with the bootstrap methods again suggested that these methods performed relatively poorly compared to the other methods (Sections 3.7 and 3.8 of the Supplementary Material), so we again omit the bootstrap methods from the main text. Figure 12 shows the coverage of 95% intervals. With normal population effects, all Q-profile intervals performed comparably to one another and showed close to nominal coverage ( 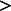 94% in 83% of scenarios). ML-profile typically showed nominal coverage or over-coverage in the large majority of scenarios; in scenarios with normal effects, the coverage of these intervals was 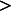 94% in 82% of scenarios, similar to the Q-profile methods. However, ML-profile did show under-coverage for smaller meta-analyses that also had high heterogeneity. This under-coverage was minimal for binary outcomes ( 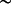 90% at minimum), but could be substantial for continuous outcomes ( 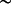 75% at minimum).

Jeffreys1-shortest showed at least nominal coverage for 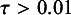 , but showed substantial under-coverage when 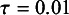 . Jeffreys2-shortest behaved similarly, except additionally showed under-coverage for meta-analyses of continuous outcomes with high heterogeneity ( 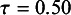 ), especially for 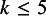 . The coverage of Jeffreys1-shortest and Jeffreys2-shortest was 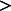 94% in, respectively, 83% and 74% of scenarios. Both Jeffreys1-central and Jeffreys2-central performed considerably worse (i.e., showed more severe under-coverage) than Jeffreys1-shortest and Jeffreys2-shortest for smaller values of 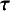 : in scenarios with normal population effects, coverage of Jeffreys1-central and Jeffreys2-central was 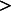 94% in, respectively, 54% and 56% of scenarios. This under-coverage reflects overestimation of 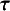 when it was near the boundary of the parameter space.

**Figure 13:**
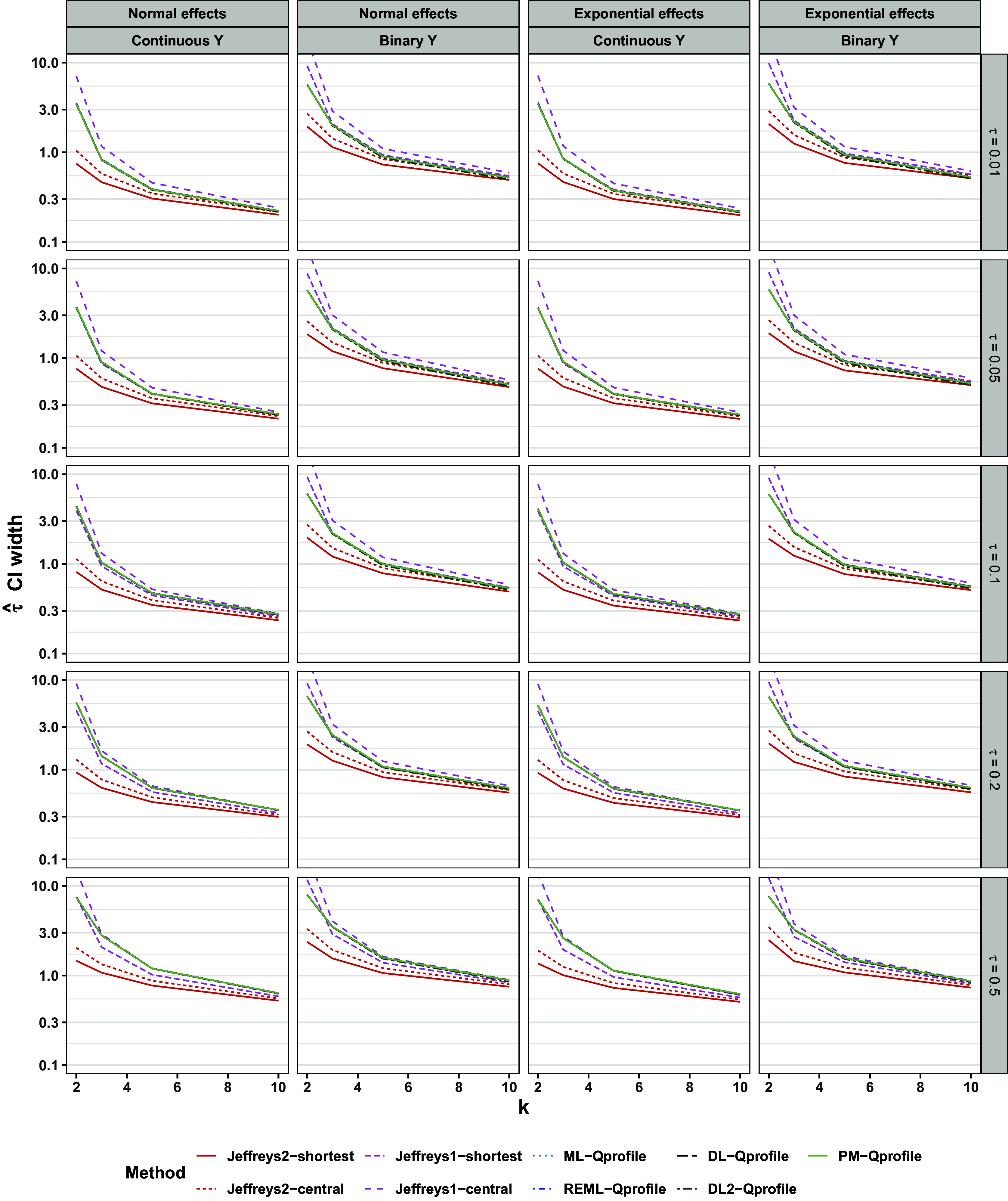
Width of CI for 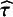 . Lines are slightly staggered horizontally for visibility. Lines are mean performances across scenarios, conditional on *k*, 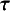 , the distribution of population effects, and the outcome type. Y-axis is on log scale.

Figure 13 shows the width of 95% intervals. We now discuss only the methods that had the highest rates of at least nominal coverage, so exclude discussion of Jeffreys2-shortest, Jeffreys1-central, and Jeffreys2-central. The widths of the various Q-profile intervals the Jeffreys1-shortest intervals were comparable, but the ML-profile intervals were typically considerably narrower, especially for very small meta-analyses.

In scenarios with exponential population effects, all methods’ relative performances for estimation and interval estimation on 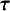 were similar, although coverages declined for all methods. Additional stratified results (Section 3 of the Supplementary Material) suggest that patterns of performance were also comparable for 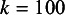 and for fixed versus varying *N* across studies.

#### Discussion of results regarding 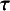


4.4.5

For point estimation of 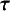 , no method emerged as clearly optimal, since methods’ performances depended strongly on 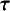 itself. The low coverage of the Jeffreys methods occurred when 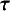 was near zero (the boundary of the parameter space). This reflects overestimation of 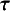 , which is often viewed as conservative in the context of random-effects meta-analysis. Regarding interval estimation for 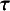 , the frequentist estimators with Q-profile or ML-profile intervals appear preferable to the Jeffreys methods. Of the two Jeffreys priors and two types of intervals, Jeffreys1-shortest is the only one whose coverage was competitive with that of the frequentist methods. However, since Jeffreys1-shortest intervals were somewhat wider than those of the frequentist intervals, this method does not seem to offer an overall advantage over the frequentist intervals. The Q-profile intervals performed slightly more consistently across scenarios than did ML-profile, although average performances were similar. ML-profile intervals were, however, considerably narrower than the Q-profile intervals.

### 4.5 Overall conclusions

All methods performed similarly for point estimation of 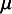 . In general, standard frequentist methods with HKSJ intervals for 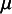 and Q-profile intervals for 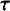 performed the most consistently across outcome types. Jeffreys2-shortest also showed consistently strong performance for meta-analyses of binary outcomes, and yielded substantially narrower intervals than frequentist methods. However, the Jeffreys2-shortest interval did not perform as consistently for continuous outcomes; this method exhibited mild under-coverage for very small meta-analyses with high heterogeneity. Regarding point estimation of 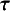 , all methods again performed comparably on average, though the optimal method depended on the value of 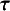 itself. Regarding interval estimation for 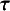 , the Q-profile method arguably performed the best and behaved consistently across scenarios.

Overall, for small meta-analyses of continuous outcomes, we would recommend standard frequentist methods with an HKSJ interval for 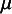 and a Q-profile interval for 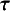 , consistent with previous recommendations. However, for small meta-analyses of binary outcomes, Jeffreys2 may be preferable over standard frequentist methods if the meta-analyst is primarily interested in point and interval estimation for 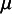 , potentially along with point estimation of 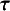 (although, again, the best-performing method for estimating 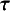 depended on the value of 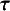 itself). This is because the Jeffreys2-shortest interval more frequently had at least nominal coverage, yet was substantially more precise. If the meta-analyst is also interested in obtaining an interval for 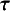 , then using a frequentist method with a Q-profile interval for 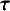 would likely provide closer to nominal coverage for 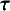 than would Jeffrey2-shortest; however, this would likely sacrifice a substantial amount of precision for 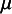 .

## 5 Applied example

Zito et al.^67^ meta-analyzed randomized trials that compared various diagnostic strategies for detecting coronary artery disease (CAD) in patients experiencing CAD-related symptoms. The authors conducted meta-analyses for each pairwise comparison between multiple diagnostic methods; for simplicity, we focus on studies comparing coronary computed tomography angiography (CCTA) to stress single-photon emission computed tomography myocardial perfusion imaging (SPECT-MPI). We replicated the authors’ meta-analyses for each of six outcomes: cardiovascular death and myocardial infarction ( 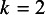 ), all-cause death ( 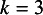 ), myocardial infarction ( 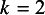 ), index invasive coronary angiography (ICA) ( 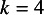 ), index revascularization ( 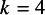 ), and downstream testing ( 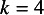 ). The authors’ meta-analyses^67^ used the DL method and used Wald rather than HKSJ confidence intervals.^iii^ We scraped study-level summary statistics from the published forest plots and re-analyzed the studies assessing each outcome using DL, REML, the exact method, Jeffreys1-shortest, and Jeffreys2-shortest. For DL and REML, we used HKSJ intervals, following established recommendations.^7^ ^,^ ^12^ ^–^ ^14^ Since our simulation study suggested relatively minor differences between the various frequentist methods with HKSJ intervals, we focus only on DL and REML for brevity. All code and data required to reproduce the applied example are publicly available and documented (https://osf.io/9qfah).

Figure 2 shows the Jeffreys1 and Jeffreys2 priors for a single outcome (all-cause death), and Figure 3 shows the resulting joint posterior under the Jeffreys2 prior. Figure 5 shows all methods’ point estimates and intervals for 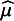 for all outcomes; a similar forest plot for heterogeneity estimates appears in Section 4 of the Supplementary Material. As in the simulation study, all point estimates were nearly identical, but the Jeffreys2-shortest interval was often considerably narrower than those from Jeffreys1-shortest, REML, DL, and the exact method. Across all six outcomes, the Jeffreys2-shortest interval on the log-odds scale was on average 45% narrower than the narrowest interval from the other methods. For the meta-analyses of only two studies, this improvement in precision increased to 112%.

## 6 Discussion

To the best of our knowledge, this paper provides the first empirical assessment of the Jeffreys2 prior in meta-analysis. We compared point estimates and intervals from the Jeffreys2 prior to those of the Jeffreys1 prior and to several of the best-performing parametric, semiparameteric, and nonparametric frequentist methods. Extending previous simulation studies on the Jeffreys1 prior, we additionally considered different types of Bayesian point estimates and intervals, and we considered point and interval estimation for both 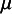 and 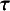 . As summarized in Section 4.5, for small meta-analyses of binary outcomes, Jeffreys2 may be preferable over standard frequentist methods for point and interval estimation for 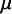 , providing improvements in efficiency that can be substantial. However, for small meta-analyses of continuous outcomes, standard frequentist methods with HKSJ intervals for 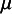 and Q-profile CIs for 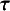 seem to be the best choices, avoiding the mild under-coverage that Jeffreys2-shortest intervals can sometimes exhibit for very small meta-analyses with high heterogeneity. For both outcome types, the best-performing method for point estimation of 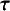 varied according to 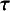 itself. When 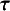 is very small, the Jeffreys methods performed conservatively in that they typically overestimate 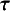 . Finally, we showed that the Jeffreys2 prior has a straightforward generalization to the case of meta-regression (Section 1 of the Supplementary Material).

Given our interest in the frequentist properties of Jeffreys priors as the Firth correction to ML estimates, we have treated point and interval estimation from a frequentist perspective. For example, our simulation study considered the coverage of 95% intervals estimated from repeated samples that were generated from fixed values of the parameters. In contrast, in Bayesian inference, the parameters are viewed as random draws from the prior, rather than as fixed quantities. The Bayesian framework does permit empirical assessment of certain analogs to coverage, but doing so involves drawing repeated samples from parameters sampled from the prior, rather than from parameters held constant.^9^ ^,^ ^68^ ^,^ ^69^ As an additional complication, performing these Bayesian calibration checks requires a proper prior from which to sample, yet both Jeffreys priors are improper.^68^ Cook et al. (2006) argued that this difficulty in assessing calibration with improper priors is a disadvantage of using such priors in the first place.^68^ Given our interest in methods’ frequentist motivations and their frequentist empirical properties, we did not consider any of the numerous other Bayesian priors that have been proposed for meta-analysis (e.g., as reviewed by Röver (2020)^9^). It is somewhat difficult to compare the performance of standard frequentist methods to Bayesian methods that lack a frequentist interpretation, which is perhaps why many previous simulation studies have not included any Bayesian methods^7^ ^,^ ^11^ (but with exceptions^15^ ^–^ ^17^).

Our simulation study had other limitations. First, we considered only one form of model misspecification, namely exponentially distributed population effects, and found that methods’ relative rankings were largely unaffected. However, we did not assess any other forms of misspecification, such as more severe departures from normality^10^ or clustered population effects. Second, for meta-analyses of binary outcomes, we considered only standard inverse-variance weighted meta-analysis, but arm-based approaches may have better statistical properties.^66^ On the other hand, arm-based methods can introduce bias due to non-exchangeability across trials,^70^ ^,^ ^71^ and inverse-variance meta-analysis more readily accommodates the possibility that studies adjust for covariates, and may be more feasible when original papers reported only limited summary statistics. Additionally, assessing inverse-variance meta-analysis provides a more direct comparison to previous simulation studies.^11^ Third, the two within-study estimators we used, namely log-odds ratios and Hedges’ *g*, both involve approximations which may introduce slight finite-sample biases of their own. Such decisions can nontrivially affect the results of simulation studies,^72^ and we used these estimators to ensure direct comparability with previous simulation studies.^7^ Additionally, these two measures are among the most commonly used in meta-analyses.^73^ Future work could explore relative performances with effect measures that do not require approximations, such as raw mean differences, although these are not frequently used in practice.^73^ Fourth, we considered only two estimands, 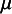 and 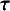 , but these alone provide a limited summary of the random-effects distribution. Additional metrics than can be informative include the percentage of population effects exceeding a chosen threshold for a meaningful effect size^47^ ^,^ ^55^ ^,^ ^74^; the prediction interval for a new population effect^54^ ^,^ ^75^; and shrinkage estimates for each study’s population effect.^75^ ^,^ ^76^ An advantage of Bayesian estimation is that such metrics can be obtained readily from the posterior; several are implemented in the R package bayesmeta.^9^ Future simulation studies could consider these estimands and intervals as well. Fifth, we made the usual assumption that any estimation error in the within-study standard errors is negligible. We did not assess the extent to which this approximation compromised interval estimation. A number of approaches have been proposed to accommodate this form of estimation error; perhaps future work could incorporate these developments into the Jeffreys priors.^77^ ^–^ ^80^

Our work remains a preliminary investigation of Jeffreys1 and Jeffreys2 priors. We would particularly encourage future work to consider other generalizations to these priors, besides our generalization to meta-regression. For example, as noted in Introduction, we recently found that a Jeffreys prior on 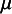 and 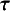 performed well for an estimation problem involving severe *p*-hacking, which required estimating the parameters of a truncated distribution.^34^ Certain selection models for publication bias lead to related distributions that involve step functions in the publication probability.^81^ These models can perform poorly for small meta-analyses, often exhibiting extremely wide intervals for parameters related to publication bias severity.^82^ ^,^ ^83^ Might using a Jeffreys prior on 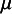 , 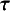 , and the bias parameters also improve these models’ performance for small meta-analyses? Additional extensions could include accommodating clustered population effects. We look forward to future research along these lines.

## Supporting information

Mathur supplementary material Mathur supplementary material

## Data Availability

All code and data required to reproduce the simulation study and applied example are publicly available and documented (https://osf.io/9qfah).

## Notes

i. The invariance property does not mean, however, that the posterior mode of  under a  -parameterization is equal to the square-root of the posterior mode of  under a  -parameterization, for example. Rather, the usual change-of-variables expression would apply.

ii. Specifically, in the context of bias correction for *p*-hacking, Mathur (2024)^34^ described meta-analyzing only studies that are nonsignificant or that have negative point estimates. In that paper, we derived the two-parameter Jeffreys prior for a version of the likelihood that is appropriately right-truncated to reflect the inclusion of only a subset of studies. The present Jeffreys2 prior is the special case in which there is no truncation, i.e., the limit as the truncation threshold approaches  .

iii. The paper states that HKSJ intervals were used, but our re-analyses replicated their results only when we used Wald intervals. Through correspondence with the authors and journal editors, we confirmed that Wald intervals were indeed used (S. Chang and J.Cornell, 14 March 2024).

## Supplementary material

The supplementary material for this article can be found at https://doi.org/10.1017/rsm.2024.2.
