# Supplementary material for: Meta-analysis with Jeffreys priors: Empirical frequentist properties
Source: Res Synth Methods. 2025 Mar 12;16(1):87–122. doi: 10.1017/rsm.2024.2 (PMC12621536; doi:10.1017/rsm.2024.2)
Supplement: Mathur supplementary material [file S1759287924000024sup001.pdf]

Supplement

Contents

1 Generalization of the Jeffreys2 prior for meta-regression 2

2 Additional figures for the simulation study 3

2.1 MAE and RMSE of $\widehat{\mu}$ 3

2.2 Comparison of posterior modes, means, and medians 5

2.3 An illustrative scenario 7

3 Additional tables for the simulation study 9

3.1 Scenarios with continuous outcomes, $k = 100$ 9

3.2 Scenarios with binary outcomes, $k = 100$ 11

3.3 Scenarios with continuous outcomes, varying N across studies 12

3.4 Scenarios with continuous outcomes, fixed N across studies 13

3.5 Scenarios with binary outcomes, varying N across studies 14

3.6 Scenarios with binary outcomes, fixed N across studies 15

3.7 Bootstrap results for scenarios with continuous outcomes, $k = 10$ 16

3.8 Bootstrap results for scenarios with binary outcomes, $k = 10$ 17

4 Additional figures for the applied example 18

1. Generalization of the Jeffreys2 prior for meta-regression

Generalizing the model given in the main text, define:

- $\boldsymbol{X}$ to be a $k \times p$ design matrix of meta-regression covariates,
- $\boldsymbol{\beta}$ to be a p-vector of meta-regression coefficients;
- $\boldsymbol{\Sigma}$ to be $\text{diag}(\sigma_1^2, \cdots, \sigma_k^2)$, the matrix of within-study variances;
- $\boldsymbol{I}_k$ to be the $k \times k$ identity matrix;
- $\boldsymbol{V}(\tau)$ to be $\tau^2 \boldsymbol{I}_k + \boldsymbol{\Sigma}$, the matrix of marginal variances;
- $S_i(\tau)$ to be $\sqrt{\tau^2 + \sigma_i^2}$, as in the main text.

Assume the point estimates are multivariate normal such that $\widehat{\boldsymbol{\theta}} \sim \text{MVN}_k\left(\boldsymbol{X}\boldsymbol{\beta}, \boldsymbol{V}(\tau)\right)$. The joint likelihood is thus:

$$\ell(\boldsymbol{\beta}, \tau) = p\left(\widehat{\boldsymbol{\theta}} \mid \boldsymbol{\beta}, \tau\right) = \frac{1}{\sqrt{(2\pi)^k \det\left(\boldsymbol{V}(\tau)\right)}} \exp\left\{ -\frac{1}{2}\left(\widehat{\boldsymbol{\theta}} - \boldsymbol{X}\boldsymbol{\beta}\right)^{\mathrm{T}} \boldsymbol{V}(\tau)^{-1} \left(\widehat{\boldsymbol{\theta}} - \boldsymbol{X}\boldsymbol{\beta}\right) \right\}$$

where $\det\left(\boldsymbol{V}(\tau)\right)$ is the determinant of $\boldsymbol{V}(\tau)$. The entries of the expected Fisher information are:

$$k_{\boldsymbol{\beta}\boldsymbol{\beta}} := E\left[\frac{\partial^2 \ell}{\partial \boldsymbol{\beta}^2}\right] = -\boldsymbol{X}^{\mathrm{T}}(\boldsymbol{V}(\tau)^{-1})\boldsymbol{X}, \quad k_{\tau\tau} := E\left[\frac{\partial^2 \ell}{\partial \tau^2}\right] = -2\tau^2 \sum_{i=1}^{k} \left(S_i(\tau)\right)^{-4}, \quad k_{\boldsymbol{\beta}\tau} := E\left[\frac{\partial^2 \ell}{\partial \boldsymbol{\beta} \partial \tau}\right] = 0$$

The Jeffreys2 prior is therefore:

$$\begin{aligned} p\left(\boldsymbol{\beta}, \tau\right) &\propto \sqrt{k_{\boldsymbol{\beta}\boldsymbol{\beta}} k_{\tau\tau}} \\ &\propto \tau \sqrt{\boldsymbol{X}^{\mathrm{T}}(\boldsymbol{V}(\tau)^{-1})\boldsymbol{X} \cdot \sum_{i=1}^{k} \left(S_i(\tau)\right)^{-4}} \end{aligned}$$

This prior leads to a proper posterior if the design matrix $\boldsymbol{X}$ is full rank, which holds if $k > p$ and there is no perfect multicollinearity. This result follows immediately from the proof of Theorem 3 in Berger (1985) (Section 4.6.3),[1] which applies to the more general case of a nonexchangeable, normal-normal hierarchical model in which the joint prior factors into an arbitrary bounded prior on τ and an independent normal prior $\boldsymbol{\beta} \sim \text{MVN}_p(\boldsymbol{\beta}^0, \boldsymbol{A})$. The hyperparameters $\boldsymbol{\beta}^0$ and $\boldsymbol{A}$ are assumed to be known. For the Jeffreys2 prior, note that $p(\boldsymbol{\beta}, \tau)$ above does not depend on $\boldsymbol{\beta}$, so can be represented as $p(\boldsymbol{\beta})\, p(\tau)$ with $p(\boldsymbol{\beta}) \propto 1$. This is a limiting case of $\boldsymbol{\beta} \sim \text{MVN}_p(\boldsymbol{\beta}^0, \boldsymbol{A})$ with $\boldsymbol{A} \to \infty$, and it can be easily seen that Berger's (1985)[1] proof holds in this limiting case. See also the discussion in Daniels (1999) (Section 3).[2]

2. Additional figures for the simulation study

2.1. MAE and RMSE of $\widehat{\mu}$

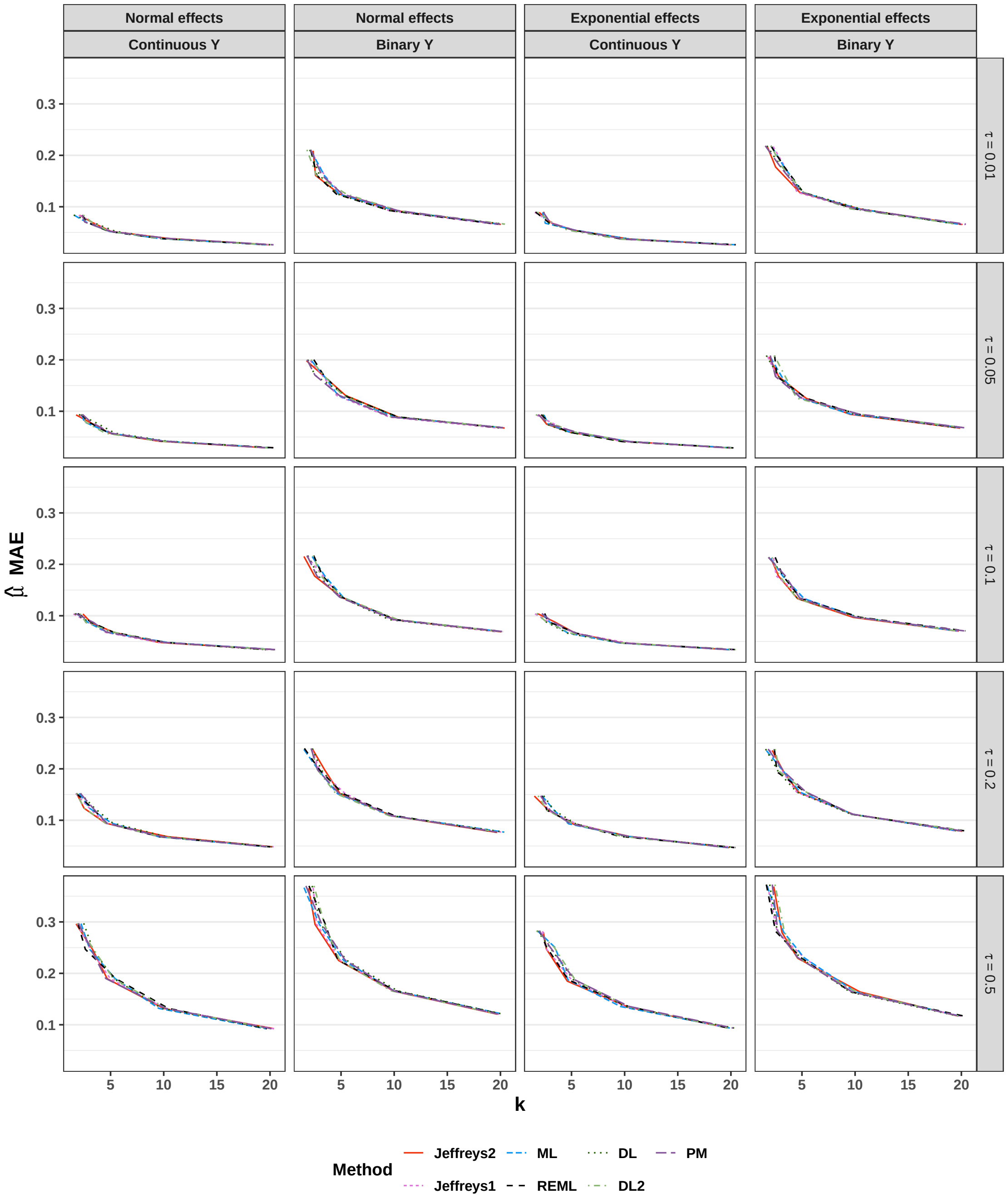

Figure S1: *MAE of $\widehat{\mu}$. Lines are slightly staggered horizontally for visibility. Lines are mean performances across scenarios, conditional on k, τ^2, the distribution of population effects, and the outcome type.*

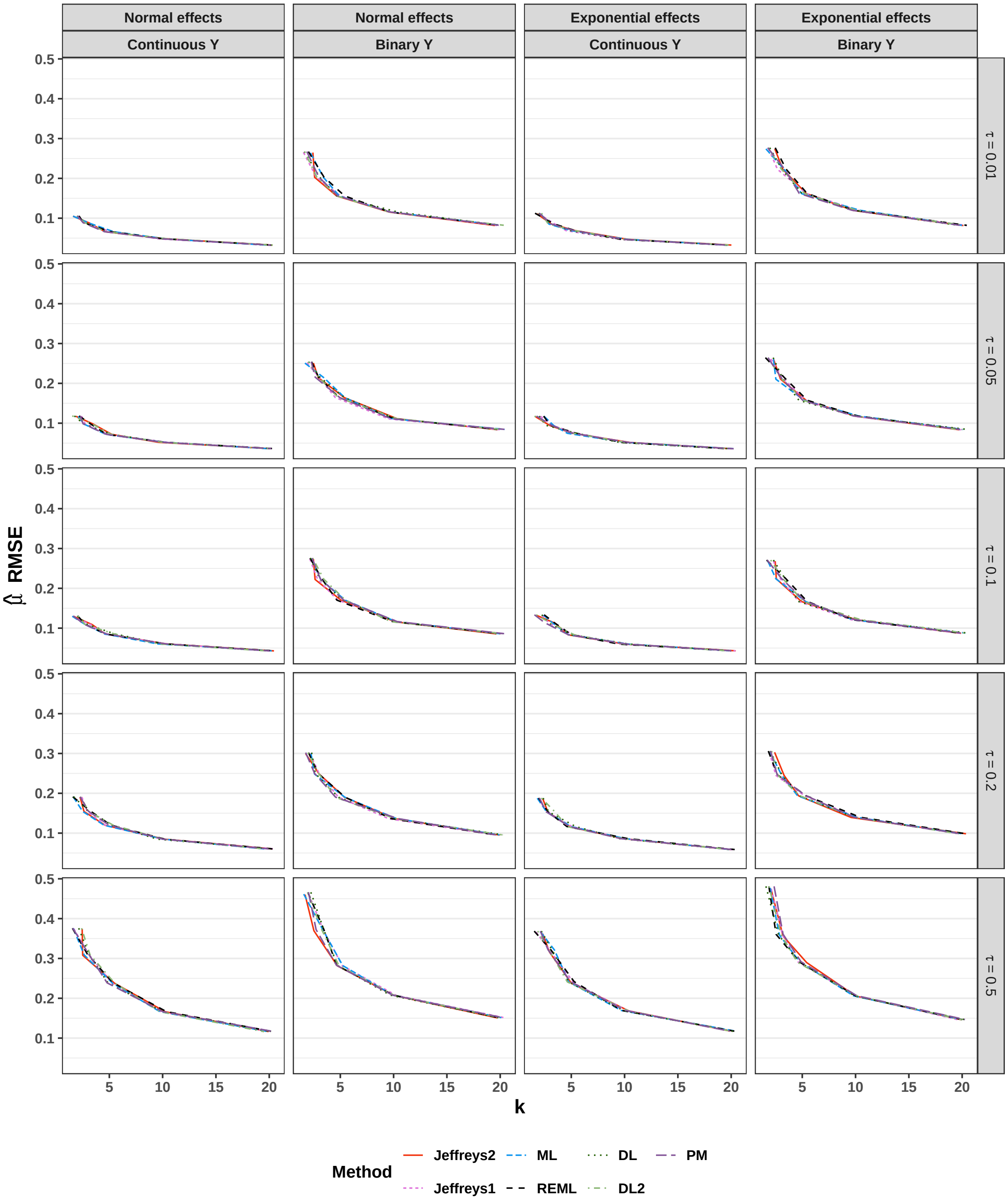

Figure S2: *RMSE of $\widehat{\mu}$. Lines are slightly staggered horizontally for visibility. Lines are mean performances across scenarios, conditional on k, τ^2, the distribution of population effects, and the outcome type.*

2.2. Comparison of posterior modes, means, and medians

The following plots compare marginal posterior modes, medians, and means under the Jeffreys2 prior for each parameter. Since the posterior of μ is usually approximately symmetric, all three measures of central tendency performed similarly. However, since the posterior of τ is usually highly skewed, the three measures performed differently. As expected theoretically, the posterior mode outperformed the other two measures.

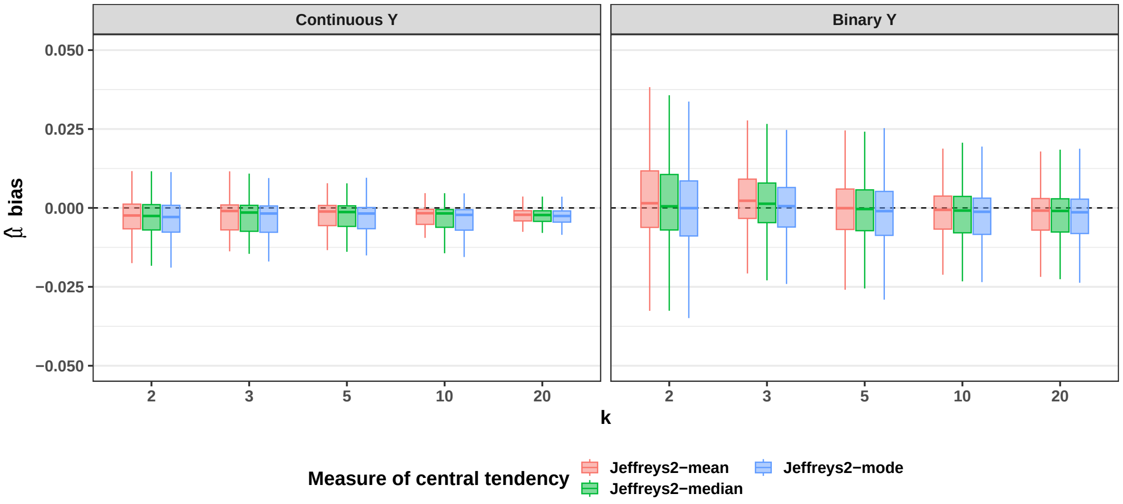

Figure S3: *Bias of $\widehat{\mu}$. Hinges of each boxplot are the 25th, 50th, and 75th percentiles. The upper and lower whiskers extend from the hinge to the minimum or maximum value that is no more than $1.5 \times$ (interquartile range) from the nearest hinge.*

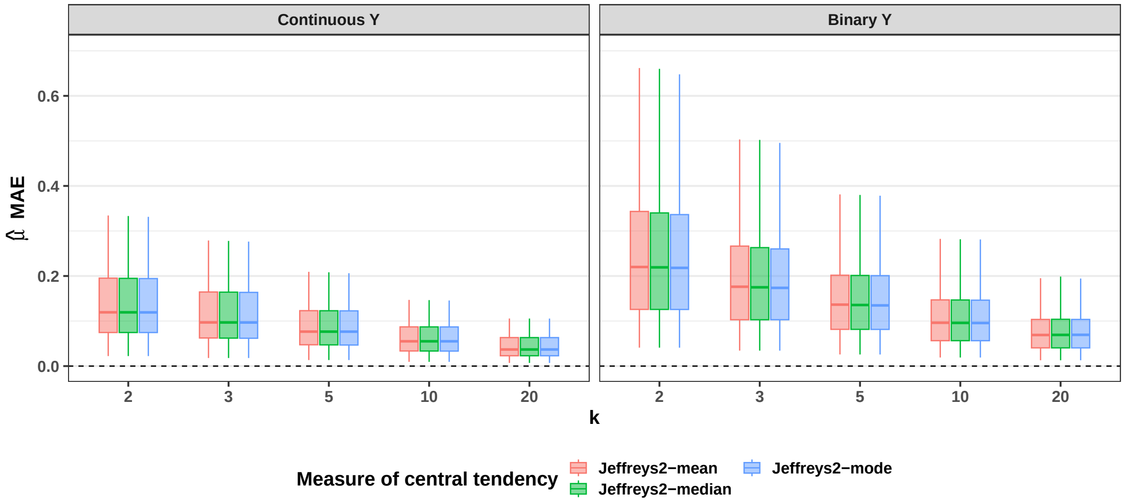

Figure S4: *MAE of $\widehat{\mu}$. Hinges of each boxplot are the 25th, 50th, and 75th percentiles. The upper and lower whiskers extend from the hinge to the minimum or maximum value that is no more than $1.5 \times$ (interquartile range) from the nearest hinge.*

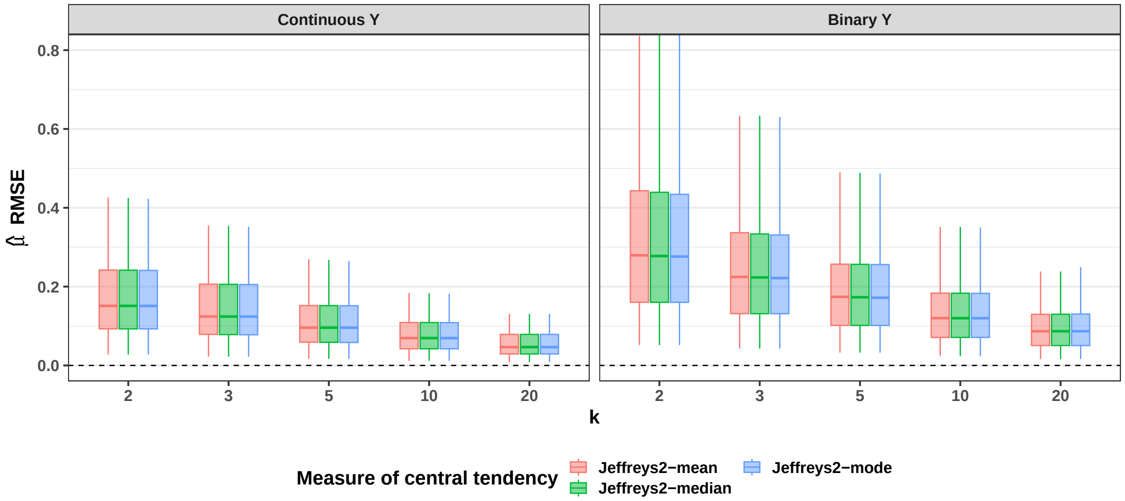

Figure S5: *RMSE of $\widehat{\mu}$. Hinges of each boxplot are the 25th, 50th, and 75th percentiles. The upper and lower whiskers extend from the hinge to the minimum or maximum value that is no more than $1.5 \times$ (interquartile range) from the nearest hinge.*

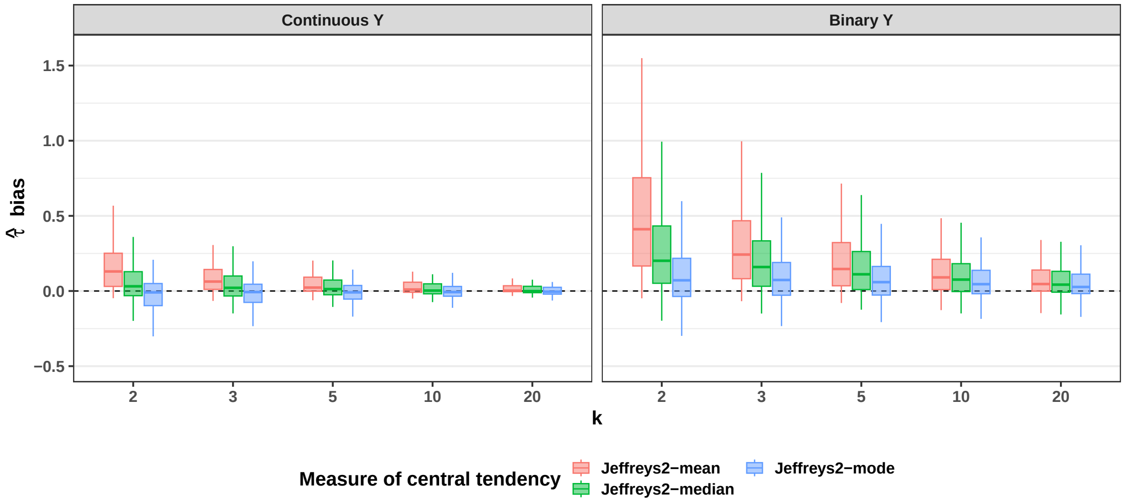

Figure S6: *Bias of $\widehat{\tau}$. Hinges of each boxplot are the 25th, 50th, and 75th percentiles. The upper and lower whiskers extend from the hinge to the minimum or maximum value that is no more than $1.5 \times$ (interquartile range) from the nearest hinge.*

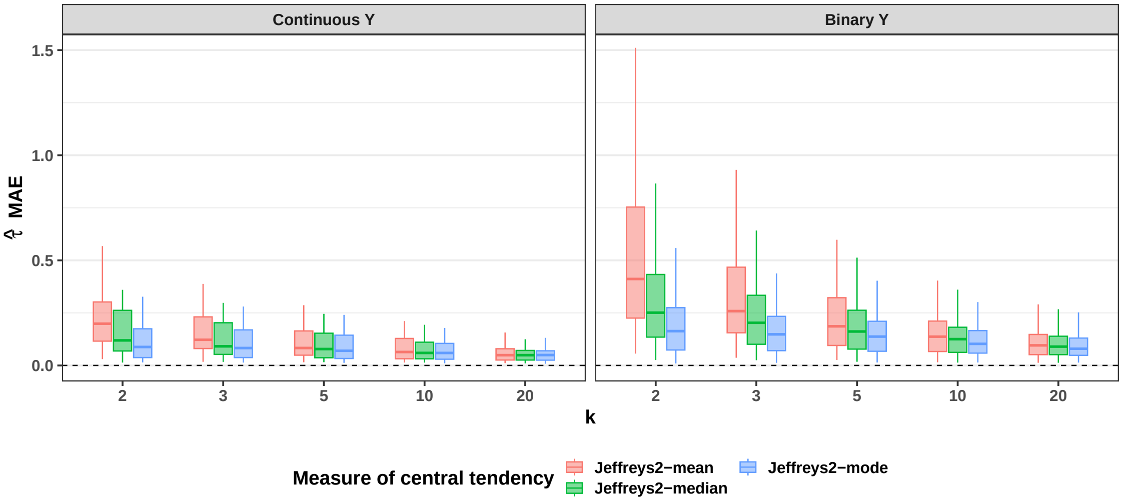

Figure S7: *MAE of $\hat{\tau}$. Hinges of each boxplot are the 25th, 50th, and 75th percentiles. The upper and lower whiskers extend from the hinge to the minimum or maximum value that is no more than* $1.5 \times$ *(interquartile range) from the nearest hinge.*

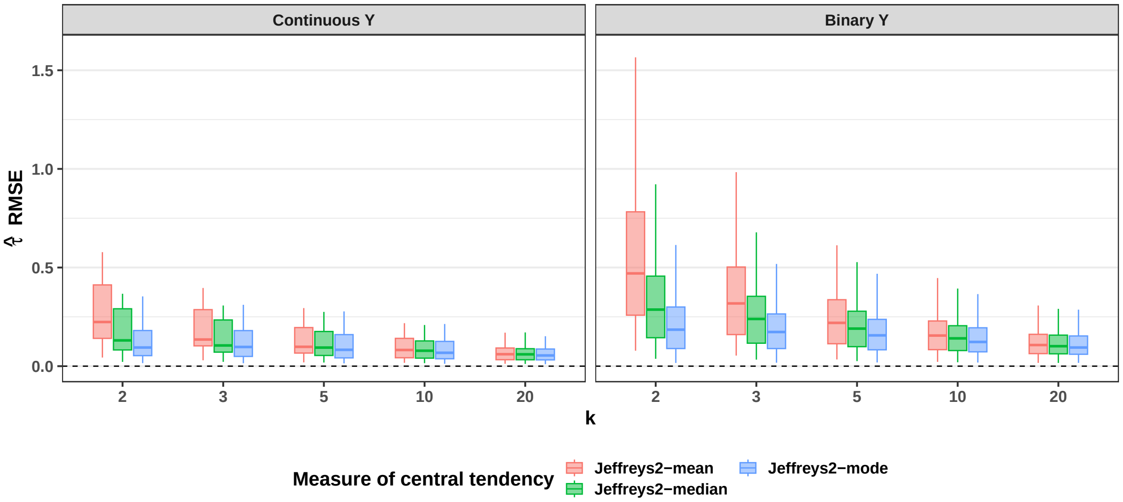

Figure S8: *RMSE of $\hat{\tau}$. Hinges of each boxplot are the 25th, 50th, and 75th percentiles. The upper and lower whiskers extend from the hinge to the minimum or maximum value that is no more than* $1.5 \times$ *(interquartile range) from the nearest hinge.*

2.3. An illustrative scenario

In this section, we isolate a single scenario selected from those in which: (1) the Jeffreys2-shortest intervals showed over-coverage, whereas the various HKSJ intervals showed under-coverage; and (2) the Jeffreys2-shortest intervals were nevertheless narrower than the HKSJ intervals. In the depicted scenario, Jeffreys2-shortest intervals had 97% coverage, whereas all

HKSJ intervals had 93% coverage. The mean width of the Jeffreys2-shortest interval was 0.81, whereas all HKSJ intervals had an average width of 1.11. The depicted scenario used the following parameters: $k = 3$, binary Y, $\mu = 0.5$, $\tau = 0.20$, normal population effects, $P(Y = 1 \mid X = 0) = 0.05$, and $N \sim U(2000, 4000)$.

Figure S9 illustrates that the width of the Jeffreys intervals varied considerably less across repeated samples than did the HKSJ intervals. Figure S10 characterizes the asymmetry of the Jeffreys2-shortest interval across repeated samples. For a given interval $[\widehat{\mu}_{lo}, \widehat{\mu}_{hi}]$ on the log-OR scale, we define the asymmetry ratio as $(\widehat{\mu}_{hi} - \widehat{\mu})/(\widehat{\mu} - \widehat{\mu}_{lo})$. Thus, an asymmetry ratio greater than 1 indicates that the upper arm of the interval is longer than the lower arm, and vice versa for an asymmetry ratio less than 1.

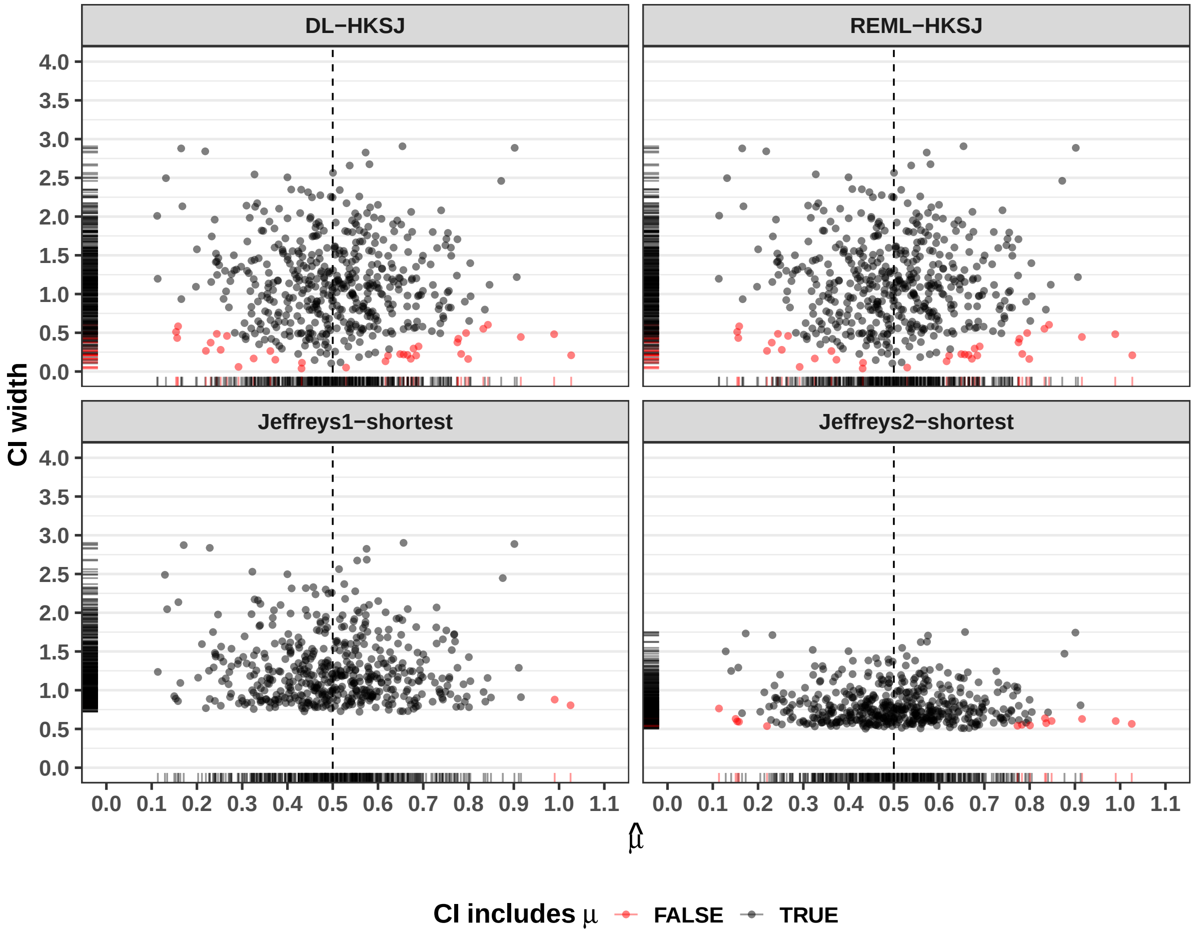

Figure S9: *Variation in $\widehat{\mu}$ and CI (credible or confidence interval) width across 500 simulation iterates (individual points). Rug plots depict marginal densities. Vertical dashed line: $\mu = 0.5$.*

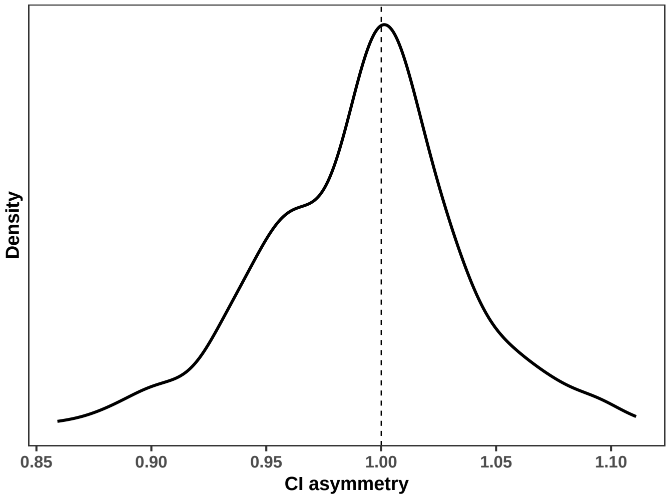

Figure S10: *Density of asymmetry ratios of Jeffreys2-shortest interval across repeated samples. For a given interval, $[\widehat{\mu}_{lo}, \widehat{\mu}_{hi}]$, the asymmetry ratio is defined as $(\widehat{\mu}_{hi} - \widehat{\mu})/(\widehat{\mu} - \widehat{\mu}_{lo})$.*

3. Additional tables for the simulation study

Tables S1 and S14 show results for scenarios with $k = 100$, which are excluded from the main text. The subsequent tables show additional stratified results for scenarios with $k \leq 20$, i.e., the same scenarios shown in the main text.

3.1. Scenarios with continuous outcomes, $k = 100$

**$\widehat{\mu}$ MAE**		**$\widehat{\mu}$ RMSE**		**$\widehat{\mu}$ coverage**		**$\widehat{\mu}$ coverage > 0.94**		**$\widehat{\mu}$ CI width**	
All	0.02	All	0.03	DL-HKSJ	0.95	Exact	0.78	All	0.10
				DL2-HKSJ	0.95	Jeffreys2-shortest	0.78		
				Exact	0.95	Jeffreys1-shortest	0.72		
				Jeffreys1-shortest	0.95	PM-HKSJ	0.70		
				Jeffreys2-shortest	0.95	DL-HKSJ	0.68		
				PM-HKSJ	0.95	DL2-HKSJ	0.68		
				REML-HKSJ	0.95	ML-HKSJ	0.68		
				ML-HKSJ	0.94	REML-HKSJ	0.68		
				ML-profile	0.94	ML-profile	0.65		

Table S1: ***Scenarios with continuous outcomes,*** $k = 100$***;*** $\widehat{\mu}$ ***point and interval estimation.*** *Methods are sorted from best to worst performance within each column (or alphabetically for ties); coverage is sorted from highest to lowest. MAE: Mean absolute error. RMSE: Root mean-square error. CI: 95% confidence or credible interval. Coverage >0.94: Percent of scenarios for which coverage probability was at least 0.94. "All": All methods performed equally to two decimal places.*

$\widehat{\tau}$ **MAE**		$\widehat{\tau}$ **RMSE**		$\widehat{\tau}$ **coverage**		$\widehat{\tau}$ **coverage** > 0.94		$\widehat{\tau}$ **CI width**	
All	0.03	Jeffreys2	0.03	DL-Qprofile	0.91	ML-profile	0.65	Jeffreys2-shortest	0.10
		DL	0.04	DL2-Qprofile	0.91	DL-Qprofile	0.58	DL-Qprofile	0.11
		DL2	0.04	ML-Qprofile	0.91	DL2-Qprofile	0.58	DL2-Qprofile	0.11
		Jeffreys1	0.04	ML-profile	0.91	ML-Qprofile	0.58	Jeffreys1-central	0.11
		ML	0.04	PM-Qprofile	0.91	PM-Qprofile	0.58	Jeffreys1-shortest	0.11
		PM	0.04	REML-Qprofile	0.91	REML-Qprofile	0.58	Jeffreys2-central	0.11
		REML	0.04	Jeffreys1-shortest	0.86	Jeffreys1-shortest	0.55	ML-Qprofile	0.11
				Jeffreys2-shortest	0.86	Jeffreys2-central	0.55	ML-profile	0.11
				Jeffreys1-central	0.84	Jeffreys1-central	0.52	PM-Qprofile	0.11
				Jeffreys2-central	0.84	Jeffreys2-shortest	0.52	REML-Qprofile	0.11

Table S2: ***Scenarios with continuous outcomes,*** $k = 100$***;*** $\widehat{\tau}$ ***point and interval estimation.*** *Methods are sorted from best to worst performance within each column (or alphabetically for ties); coverage is sorted from highest to lowest. MAE: Mean absolute error. RMSE: Root mean-square error. CI: 95% confidence or credible interval. Coverage >0.94: Percent of scenarios for which coverage probability was at least 0.94. "All": All methods performed equally to two decimal places.*

3.2. Scenarios with binary outcomes, $k = 100$

$\hat{\mu}$ **MAE**		$\hat{\mu}$ **RMSE**		$\hat{\mu}$ **coverage**		$\hat{\mu}$ **coverage** > 0.94		$\hat{\mu}$ **CI width**	
All	0.04	All	0.05	Jeffreys1-shortest	0.94	Jeffreys1-shortest	0.76	DL-HKSJ	0.17
				Jeffreys2-shortest	0.93	Jeffreys2-shortest	0.74	DL2-HKSJ	0.17
				ML-profile	0.93	ML-profile	0.63	ML-HKSJ	0.17
				DL-HKSJ	0.92	DL-HKSJ	0.60	PM-HKSJ	0.17
				DL2-HKSJ	0.92	DL2-HKSJ	0.60	REML-HKSJ	0.17
				ML-HKSJ	0.92	PM-HKSJ	0.60	Jeffreys1-shortest	0.18
				PM-HKSJ	0.92	REML-HKSJ	0.60	Jeffreys2-shortest	0.18
				REML-HKSJ	0.92	ML-HKSJ	0.59	ML-profile	0.18
				Exact		Exact		Exact	

Table S3: ***Scenarios with binary outcomes,*** $k = 100$***;*** $\hat{\mu}$ ***point and interval estimation.*** *Methods are sorted from best to worst performance within each column (or alphabetically for ties); coverage is sorted from highest to lowest. MAE: Mean absolute error. RMSE: Root mean-square error. CI: 95% confidence or credible interval. Coverage >0.94: Percent of scenarios for which coverage probability was at least 0.94. "All": All methods performed equally to two decimal places.*

$\hat{\tau}$ **MAE**		$\hat{\tau}$ **RMSE**		$\hat{\tau}$ **coverage**		$\hat{\tau}$ **coverage** > 0.94		$\hat{\tau}$ **CI width**	
All	0.06	Jeffreys2	0.06	ML-profile	0.93	ML-profile	0.67	DL2-Qprofile	0.17
		DL	0.07	DL-Qprofile	0.86	DL-Qprofile	0.58	DL-Qprofile	0.18
		DL2	0.07	ML-Qprofile	0.86	ML-Qprofile	0.58	ML-Qprofile	0.18
		Jeffreys1	0.07	PM-Qprofile	0.86	PM-Qprofile	0.58	PM-Qprofile	0.18
		ML	0.07	REML-Qprofile	0.86	REML-Qprofile	0.58	REML-Qprofile	0.18
		PM	0.07	DL2-Qprofile	0.85	Jeffreys1-central	0.54	Jeffreys1-shortest	0.20
		REML	0.07	Jeffreys2-shortest	0.83	Jeffreys1-shortest	0.54	Jeffreys2-central	0.20
				Jeffreys1-shortest	0.82	DL2-Qprofile	0.53	Jeffreys2-shortest	0.20
				Jeffreys1-central	0.79	Jeffreys2-central	0.53	ML-profile	0.20
				Jeffreys2-central	0.79	Jeffreys2-shortest	0.53	Jeffreys1-central	0.21

Table S4: ***Scenarios with binary outcomes,*** $k = 100$***;*** $\hat{\tau}$ ***point and interval estimation.*** *Methods are sorted from best to worst performance within each column (or alphabetically for ties); coverage is sorted from highest to lowest. MAE: Mean absolute error. RMSE: Root mean-square error. CI: 95% confidence or credible interval. Coverage >0.94: Percent of scenarios for which coverage probability was at least 0.94. "All": All methods performed equally to two decimal places.*

3.3. Scenarios with continuous outcomes, varying N across studies

$\widehat{\mu}$ **MAE**		$\widehat{\mu}$ **RMSE**		$\widehat{\mu}$ **coverage**		$\widehat{\mu}$ **coverage** > 0.94		$\widehat{\mu}$ **CI width**	
All	0.08	All	0.10	Jeffreys1-shortest	0.97	Jeffreys1-shortest	0.85	ML-profile	0.41
				Exact	0.96	Exact	0.80	Jeffreys2-shortest	0.51
				Jeffreys2-shortest	0.95	PM-HKSJ	0.67	ML-HKSJ	0.99
				DL-HKSJ	0.94	REML-HKSJ	0.67	DL-HKSJ	1.00
				DL2-HKSJ	0.94	DL-HKSJ	0.66	DL2-HKSJ	1.00
				ML-HKSJ	0.94	DL2-HKSJ	0.66	PM-HKSJ	1.00
				PM-HKSJ	0.94	Jeffreys2-shortest	0.66	REML-HKSJ	1.00
				REML-HKSJ	0.94	ML-HKSJ	0.66	Exact	1.08
				ML-profile	0.91	ML-profile	0.40	Jeffreys1-shortest	1.20

Table S5: ***Scenarios with continuous outcomes, varying*** N ***across studies;*** $\widehat{\mu}$ ***point and interval estimation.*** *Methods are sorted from best to worst performance within each column (or alphabetically for ties); coverage is sorted from highest to lowest. MAE: Mean absolute error. RMSE: Root mean-square error. CI: 95% confidence or credible interval. Coverage >0.94: Percent of scenarios for which coverage probability was at least 0.94. "All": All methods performed equally to two decimal places.*

$\widehat{\tau}$ **MAE**		$\widehat{\tau}$ **RMSE**		$\widehat{\tau}$ **coverage**		$\widehat{\tau}$ **coverage** > 0.94		$\widehat{\tau}$ **CI width**	
Jeffreys2	0.07	Jeffreys1	0.09	Jeffreys1-shortest	0.95	Jeffreys1-shortest	0.74	ML-profile	0.33
DL	0.08	Jeffreys2	0.09	DL-Qprofile	0.92	Jeffreys2-central	0.60	Jeffreys2-shortest	0.37
DL2	0.08	DL	0.10	DL2-Qprofile	0.92	Jeffreys1-central	0.58	Jeffreys2-central	0.46
Jeffreys1	0.08	DL2	0.10	ML-Qprofile	0.92	Jeffreys2-shortest	0.55	Jeffreys1-shortest	1.04
ML	0.08	ML	0.10	PM-Qprofile	0.92	DL2-Qprofile	0.51	DL2-Qprofile	1.25
PM	0.08	PM	0.10	REML-Qprofile	0.92	DL-Qprofile	0.49	DL-Qprofile	1.26
REML	0.08	REML	0.10	Jeffreys2-shortest	0.91	ML-Qprofile	0.49	ML-Qprofile	1.26
				ML-profile	0.89	ML-profile	0.49	PM-Qprofile	1.26
				Jeffreys2-central	0.84	PM-Qprofile	0.49	REML-Qprofile	1.26
				Jeffreys1-central	0.83	REML-Qprofile	0.49	Jeffreys1-central	1.82

Table S6: ***Scenarios with continuous outcomes, varying*** N ***across studies;*** $\widehat{\tau}$ ***point and interval estimation.*** *Methods are sorted from best to worst performance within each column (or alphabetically for ties); coverage is sorted from highest to lowest. MAE: Mean absolute error. RMSE: Root mean-square error. CI: 95% confidence or credible interval. Coverage >0.94: Percent of scenarios for which coverage probability was at least 0.94.*

3.4. Scenarios with continuous outcomes, fixed N across studies

$\widehat{\mu}$ **MAE**		$\widehat{\mu}$ **RMSE**		$\widehat{\mu}$ **coverage**		$\widehat{\mu}$ **coverage** > 0.94		$\widehat{\mu}$ **CI width**	
All	0.11	All	0.14	Jeffreys1-shortest	0.98	Jeffreys1-shortest	0.93	ML-profile	0.61
				Exact	0.97	Exact	0.88	Jeffreys2-shortest	0.83
				Jeffreys2-shortest	0.97	Jeffreys2-shortest	0.79	DL-HKSJ	1.39
				DL-HKSJ	0.94	DL-HKSJ	0.62	DL2-HKSJ	1.39
				DL2-HKSJ	0.94	DL2-HKSJ	0.62	ML-HKSJ	1.39
				ML-HKSJ	0.94	ML-HKSJ	0.62	PM-HKSJ	1.39
				ML-profile	0.94	REML-HKSJ	0.62	REML-HKSJ	1.39
				PM-HKSJ	0.94	PM-HKSJ	0.61	Exact	1.63
				REML-HKSJ	0.94	ML-profile	0.60	Jeffreys1-shortest	1.93

Table S7: ***Scenarios with continuous outcomes, fixed*** N ***across studies;*** $\widehat{\mu}$ ***point and interval estimation.*** *Methods are sorted from best to worst performance within each column (or alphabetically for ties); coverage is sorted from highest to lowest. MAE: Mean absolute error. RMSE: Root mean-square error. CI: 95% confidence or credible interval. Coverage >0.94: Percent of scenarios for which coverage probability was at least 0.94. "All": All methods performed equally to two decimal places.*

$\widehat{\tau}$ **MAE**		$\widehat{\tau}$ **RMSE**		$\widehat{\tau}$ **coverage**		$\widehat{\tau}$ **coverage** > 0.94		$\widehat{\tau}$ **CI width**	
Jeffreys2	0.10	Jeffreys2	0.12	ML-profile	0.95	Jeffreys1-shortest	0.75	ML-profile	0.52
DL	0.11	Jeffreys1	0.13	DL-Qprofile	0.94	ML-profile	0.74	Jeffreys2-shortest	0.62
DL2	0.11	ML	0.13	DL2-Qprofile	0.94	Jeffreys2-shortest	0.68	Jeffreys2-central	0.77
Jeffreys1	0.11	DL	0.14	ML-Qprofile	0.94	DL-Qprofile	0.64	DL-Qprofile	1.69
ML	0.11	DL2	0.15	PM-Qprofile	0.94	ML-Qprofile	0.64	DL2-Qprofile	1.69
PM	0.11	PM	0.15	REML-Qprofile	0.94	PM-Qprofile	0.64	ML-Qprofile	1.69
REML	0.11	REML	0.15	Jeffreys1-shortest	0.92	REML-Qprofile	0.64	PM-Qprofile	1.69
				Jeffreys2-shortest	0.91	DL2-Qprofile	0.62	REML-Qprofile	1.69
				Jeffreys2-central	0.74	Jeffreys2-central	0.56	Jeffreys1-shortest	1.70
				Jeffreys1-central	0.71	Jeffreys1-central	0.54	Jeffreys1-central	2.97

Table S8: ***Scenarios with continuous outcomes, fixed*** N ***across studies;*** $\widehat{\tau}$ ***point and interval estimation.*** *Methods are sorted from best to worst performance within each column (or alphabetically for ties); coverage is sorted from highest to lowest. MAE: Mean absolute error. RMSE: Root mean-square error. CI: 95% confidence or credible interval. Coverage >0.94: Percent of scenarios for which coverage probability was at least 0.94.*

3.5. Scenarios with binary outcomes, varying N across studies

$\widehat{\mu}$ **MAE**		$\widehat{\mu}$ **RMSE**		$\widehat{\mu}$ **coverage**		$\widehat{\mu}$ **coverage** > 0.94		$\widehat{\mu}$ **CI width**	
All	0.12	Jeffreys1	0.15	Exact	0.98	Jeffreys1-shortest	0.93	ML-profile	0.71
		Jeffreys2	0.15	Jeffreys1-shortest	0.98	Exact	0.91	Jeffreys2-shortest	0.98
		ML	0.15	Jeffreys2-shortest	0.97	Jeffreys2-shortest	0.83	ML-HKSJ	1.58
		DL	0.16	DL-HKSJ	0.95	DL-HKSJ	0.72	DL-HKSJ	1.62
		DL2	0.16	DL2-HKSJ	0.95	DL2-HKSJ	0.72	DL2-HKSJ	1.62
		PM	0.16	ML-HKSJ	0.95	ML-HKSJ	0.72	PM-HKSJ	1.62
		REML	0.16	PM-HKSJ	0.95	PM-HKSJ	0.72	REML-HKSJ	1.62
				REML-HKSJ	0.95	REML-HKSJ	0.72	Exact	1.94
				ML-profile	0.94	ML-profile	0.64	Jeffreys1-shortest	2.34

Table S9: ***Scenarios with binary outcomes, varying N across studies; $\widehat{\mu}$ point and interval estimation.*** *Methods are sorted from best to worst performance within each column (or alphabetically for ties); coverage is sorted from highest to lowest. MAE: Mean absolute error. RMSE: Root mean-square error. CI: 95% confidence or credible interval. Coverage >0.94: Percent of scenarios for which coverage probability was at least 0.94. "All": All methods performed equally to two decimal places.*

$\widehat{\tau}$ **MAE**		$\widehat{\tau}$ **RMSE**		$\widehat{\tau}$ **coverage**		$\widehat{\tau}$ **coverage** > 0.94		$\widehat{\tau}$ **CI width**	
Jeffreys2	0.11	Jeffreys2	0.13	ML-profile	0.95	Jeffreys1-shortest	0.78	ML-profile	0.59
ML	0.11	ML	0.14	DL-Qprofile	0.94	ML-profile	0.73	Jeffreys2-shortest	0.73
DL	0.12	Jeffreys1	0.15	DL2-Qprofile	0.94	DL-Qprofile	0.70	Jeffreys2-central	0.92
REML	0.12	DL	0.16	ML-Qprofile	0.94	DL2-Qprofile	0.70	DL2-Qprofile	1.85
DL2	0.13	REML	0.16	PM-Qprofile	0.94	Jeffreys2-shortest	0.70	DL-Qprofile	1.87
Jeffreys1	0.13	DL2	0.17	REML-Qprofile	0.94	ML-Qprofile	0.70	ML-Qprofile	1.87
PM	0.13	PM	0.17	Jeffreys1-shortest	0.93	PM-Qprofile	0.70	PM-Qprofile	1.87
				Jeffreys2-shortest	0.91	REML-Qprofile	0.70	REML-Qprofile	1.87
				Jeffreys2-central	0.73	Jeffreys2-central	0.55	Jeffreys1-shortest	2.06
				Jeffreys1-central	0.70	Jeffreys1-central	0.51	Jeffreys1-central	3.63

Table S10: ***Scenarios with binary outcomes, varying N across studies; $\widehat{\tau}$ point and interval estimation.*** *Methods are sorted from best to worst performance within each column (or alphabetically for ties); coverage is sorted from highest to lowest. MAE: Mean absolute error. RMSE: Root mean-square error. CI: 95% confidence or credible interval. Coverage >0.94: Percent of scenarios for which coverage probability was at least 0.94.*

3.6. Scenarios with binary outcomes, fixed N across studies

$\widehat{\mu}$ **MAE**		$\widehat{\mu}$ **RMSE**		$\widehat{\mu}$ **coverage**		$\widehat{\mu}$ **coverage** > 0.94		$\widehat{\mu}$ **CI width**	
All	0.20	All	0.25	Jeffreys1-shortest	0.99	Jeffreys1-shortest	0.98	ML-profile	1.26
				Jeffreys2-shortest	0.99	Exact	0.96	Jeffreys2-shortest	1.85
				Exact	0.98	Jeffreys2-shortest	0.96	ML-HKSJ	2.60
				ML-profile	0.96	ML-profile	0.84	DL-HKSJ	2.62
				DL-HKSJ	0.95	DL-HKSJ	0.75	DL2-HKSJ	2.62
				DL2-HKSJ	0.95	DL2-HKSJ	0.75	PM-HKSJ	2.62
				ML-HKSJ	0.95	ML-HKSJ	0.75	REML-HKSJ	2.62
				PM-HKSJ	0.95	PM-HKSJ	0.75	Exact	3.46
				REML-HKSJ	0.95	REML-HKSJ	0.75	Jeffreys1-shortest	4.35

Table S11: ***Scenarios with binary outcomes, fixed*** N ***across studies;*** $\widehat{\mu}$ ***point and interval estimation.*** *Methods are sorted from best to worst performance within each column (or alphabetically for ties); coverage is sorted from highest to lowest. MAE: Mean absolute error. RMSE: Root mean-square error. CI: 95% confidence or credible interval. Coverage >0.94: Percent of scenarios for which coverage probability was at least 0.94. "All": All methods performed equally to two decimal places.*

$\widehat{\tau}$ **MAE**		$\widehat{\tau}$ **RMSE**		$\widehat{\tau}$ **coverage**		$\widehat{\tau}$ **coverage** > 0.94		$\widehat{\tau}$ **CI width**	
ML	0.14	ML	0.18	ML-profile	0.98	ML-profile	0.91	ML-profile	1.05
DL	0.17	DL	0.23	DL-Qprofile	0.94	Jeffreys1-shortest	0.77	Jeffreys2-shortest	1.37
DL2	0.17	Jeffreys2	0.23	DL2-Qprofile	0.94	Jeffreys2-shortest	0.74	Jeffreys2-central	1.72
REML	0.17	DL2	0.24	ML-Qprofile	0.94	DL-Qprofile	0.73	DL2-Qprofile	2.56
PM	0.18	PM	0.24	PM-Qprofile	0.94	ML-Qprofile	0.73	DL-Qprofile	2.58
Jeffreys2	0.21	REML	0.24	REML-Qprofile	0.94	PM-Qprofile	0.73	ML-Qprofile	2.58
Jeffreys1	0.25	Jeffreys1	0.27	Jeffreys1-shortest	0.87	REML-Qprofile	0.73	PM-Qprofile	2.58
				Jeffreys2-shortest	0.86	DL2-Qprofile	0.71	REML-Qprofile	2.58
				Jeffreys2-central	0.62	Jeffreys2-central	0.47	Jeffreys1-shortest	3.82
				Jeffreys1-central	0.57	Jeffreys1-central	0.39	Jeffreys1-central	6.76

Table S12: ***Scenarios with binary outcomes, fixed*** N ***across studies;*** $\widehat{\tau}$ ***point and interval estimation.*** *Methods are sorted from best to worst performance within each column (or alphabetically for ties); coverage is sorted from highest to lowest. MAE: Mean absolute error. RMSE: Root mean-square error. CI: 95% confidence or credible interval. Coverage >0.94: Percent of scenarios for which coverage probability was at least 0.94.*

3.7. Bootstrap results for scenarios with continuous outcomes, $k = 10$

As noted in the main text, the BCa bootstrap only provided an interval for $\widehat{\mu}$ and $\widehat{\tau}$ in 67% of datasets. Given this low convergence rate, some caution is warranted when comparing this method's performance to that of other methods.

$\widehat{\mu}$ **MAE**		$\widehat{\mu}$ **RMSE**		$\widehat{\mu}$ **coverage**		$\widehat{\mu}$ **coverage** > 0.94		$\widehat{\mu}$ **CI width**	
Jeffreys1	0.06	All	0.08	Exact	0.96	Jeffreys1-shortest	0.80	Boot-perc	0.32
Jeffreys2	0.06			Jeffreys1-shortest	0.96	DL-HKSJ	0.78	Boot-BCa	0.33
DL	0.07			Jeffreys2-shortest	0.96	DL2-HKSJ	0.78	ML-profile	0.33
DL2	0.07			DL-HKSJ	0.94	Exact	0.78	DL-HKSJ	0.36
ML	0.07			DL2-HKSJ	0.94	ML-HKSJ	0.78	DL2-HKSJ	0.36
PM	0.07			ML-HKSJ	0.94	PM-HKSJ	0.78	Exact	0.36
REML	0.07			ML-profile	0.94	REML-HKSJ	0.78	Jeffreys2-shortest	0.36
				PM-HKSJ	0.94	Jeffreys2-shortest	0.70	ML-HKSJ	0.36
				REML-HKSJ	0.94	ML-profile	0.55	PM-HKSJ	0.36
				Boot-perc	0.93	Boot-perc	0.42	REML-HKSJ	0.36
				Boot-BCa	0.92	Boot-BCa	0.32	Jeffreys1-shortest	0.38

Table S13: ***Bootstrap results for scenarios with continuous outcomes,*** $k = 10$***;*** $\widehat{\mu}$ ***point and interval estimation.*** *Methods are sorted from best to worst performance within each column (or alphabetically for ties); coverage is sorted from highest to lowest. MAE: Mean absolute error. RMSE: Root mean-square error. CI: 95% confidence or credible interval. Coverage >0.94: Percent of scenarios for which coverage probability was at least 0.94. "All": All methods performed equally to two decimal places.*

$\widehat{\tau}$ **MAE**		$\widehat{\tau}$ **RMSE**		$\widehat{\tau}$ **coverage**		$\widehat{\tau}$ **coverage** > 0.94		$\widehat{\tau}$ **CI width**	
All	0.07	Jeffreys2	0.08	ML-profile	0.93	DL-Qprofile	0.65	Boot-perc	0.26
		DL	0.09	DL-Qprofile	0.92	ML-Qprofile	0.65	Boot-BCa	0.27
		DL2	0.09	DL2-Qprofile	0.92	PM-Qprofile	0.65	Jeffreys2-shortest	0.29
		Jeffreys1	0.09	ML-Qprofile	0.92	REML-Qprofile	0.65	ML-profile	0.29
		ML	0.09	PM-Qprofile	0.92	DL2-Qprofile	0.62	Jeffreys2-central	0.31
		PM	0.09	REML-Qprofile	0.92	Jeffreys1-shortest	0.60	Jeffreys1-shortest	0.32
		REML	0.09	Jeffreys1-shortest	0.88	ML-profile	0.58	DL-Qprofile	0.34
				Jeffreys2-shortest	0.88	Boot-perc	0.50	DL2-Qprofile	0.34
				Boot-BCa	0.87	Jeffreys2-central	0.50	ML-Qprofile	0.34
				Boot-perc	0.87	Jeffreys2-shortest	0.50	PM-Qprofile	0.34
				Jeffreys1-central	0.79	Jeffreys1-central	0.48	REML-Qprofile	0.34
				Jeffreys2-central	0.79	Boot-BCa	0.20	Jeffreys1-central	0.35

Table S14: ***Bootstrap results for scenarios with continuous outcomes,*** $k = 10$***;*** $\widehat{\tau}$ ***point and interval estimation.*** *Methods are sorted from best to worst performance within each column (or alphabetically for ties); coverage is sorted from highest to lowest. MAE: Mean absolute error. RMSE: Root mean-square error. CI: 95% confidence or credible interval. Coverage >0.94: Percent of scenarios for which coverage probability was at least 0.94. "All": All methods performed equally to two decimal places.*

3.8. Bootstrap results for scenarios with binary outcomes, $k = 10$

$\widehat{\mu}$ **MAE**		$\widehat{\mu}$ **RMSE**		$\widehat{\mu}$ **coverage**		$\widehat{\mu}$ **coverage** > 0.94		$\widehat{\mu}$ **CI width**	
All	0.12	All	0.15	Jeffreys1-shortest	0.98	Jeffreys1-shortest	0.97	Boot-perc	0.63
				Jeffreys2-shortest	0.98	Exact	0.95	Boot-BCa	0.65
				Exact	0.97	Jeffreys2-shortest	0.89	ML-profile	0.67
				ML-profile	0.96	DL-HKSJ	0.84	ML-HKSJ	0.68
				DL-HKSJ	0.95	DL2-HKSJ	0.84	DL-HKSJ	0.69
				DL2-HKSJ	0.95	PM-HKSJ	0.84	DL2-HKSJ	0.69
				ML-HKSJ	0.95	REML-HKSJ	0.84	PM-HKSJ	0.69
				PM-HKSJ	0.95	ML-HKSJ	0.82	REML-HKSJ	0.69
				REML-HKSJ	0.95	ML-profile	0.76	Exact	0.75
				Boot-BCa	0.94	Boot-BCa	0.58	Jeffreys2-shortest	0.77
				Boot-perc	0.94	Boot-perc	0.53	Jeffreys1-shortest	0.81

Table S15: ***Bootstrap results for scenarios with binary outcomes,*** $k = 10$***;*** $\widehat{\mu}$ ***point and interval estimation.*** *Methods are sorted from best to worst performance within each column (or alphabetically for ties); coverage is sorted from highest to lowest. MAE: Mean absolute error. RMSE: Root mean-square error. CI: 95% confidence or credible interval. Coverage >0.94: Percent of scenarios for which coverage probability was at least 0.94. "All": All methods performed equally to two decimal places.*

$\widehat{\tau}$ **MAE**		$\widehat{\tau}$ **RMSE**		$\widehat{\tau}$ **coverage**		$\widehat{\tau}$ **coverage** > 0.94		$\widehat{\tau}$ **CI width**	
ML	0.12	ML	0.15	ML-profile	0.97	DL-Qprofile	0.82	Boot-perc	0.49
DL	0.13	DL	0.16	DL-Qprofile	0.95	DL2-Qprofile	0.82	Boot-BCa	0.57
DL2	0.13	Jeffreys2	0.16	DL2-Qprofile	0.95	ML-Qprofile	0.82	ML-profile	0.60
PM	0.13	REML	0.16	ML-Qprofile	0.95	PM-Qprofile	0.82	Jeffreys2-shortest	0.64
REML	0.13	DL2	0.17	PM-Qprofile	0.95	REML-Qprofile	0.82	Jeffreys2-central	0.68
Jeffreys2	0.14	PM	0.17	REML-Qprofile	0.95	ML-profile	0.76	DL2-Qprofile	0.70
Jeffreys1	0.16	Jeffreys1	0.18	Boot-BCa	0.92	Jeffreys1-shortest	0.68	Jeffreys1-shortest	0.71
				Boot-perc	0.92	Jeffreys2-shortest	0.63	DL-Qprofile	0.74
				Jeffreys1-shortest	0.82	Boot-perc	0.61	ML-Qprofile	0.74
				Jeffreys2-shortest	0.82	Jeffreys2-central	0.53	PM-Qprofile	0.74
				Jeffreys2-central	0.72	Jeffreys1-central	0.50	REML-Qprofile	0.74
				Jeffreys1-central	0.69	Boot-BCa	0.34	Jeffreys1-central	0.76

Table S16: ***Bootstrap results for scenarios with binary outcomes,*** $k = 10$***;*** $\widehat{\tau}$ ***point and interval estimation.*** *Methods are sorted from best to worst performance within each column (or alphabetically for ties); coverage is sorted from highest to lowest. MAE: Mean absolute error. RMSE: Root mean-square error. CI: 95% confidence or credible interval. Coverage >0.94: Percent of scenarios for which coverage probability was at least 0.94. "All": All methods performed equally to two decimal places.*

4. Additional figures for the applied example

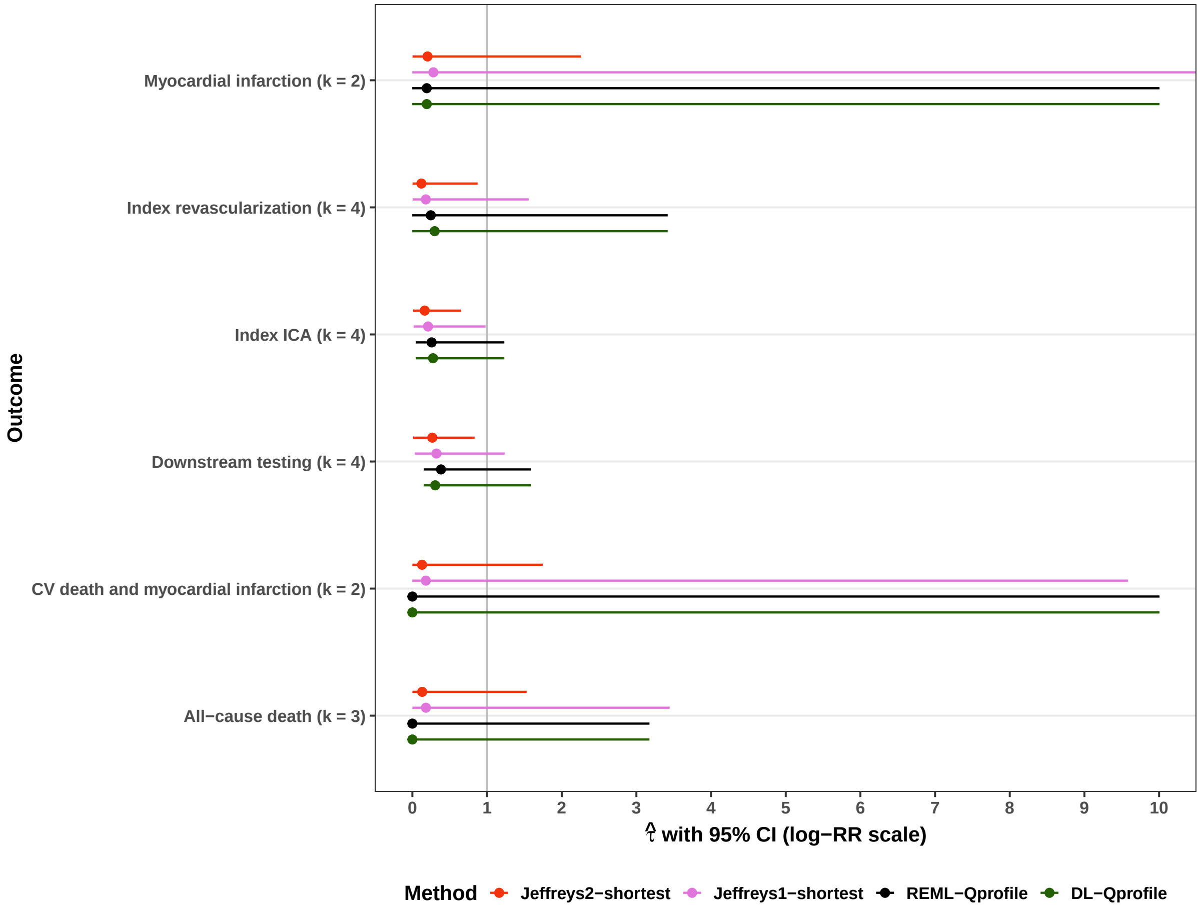

Figure S11: *Interval limits greater than* $\log(RR) = 10$ *are truncated.*

References

1. Berger JO. Bayesian Analysis. Statistical Decision Theory: Foundations, Concepts, and Methods 1985 :89–168

2. Daniels MJ. A prior for the variance in hierarchical models. Canadian Journal of Statistics 1999; 27:567–78
